# Climate-driven host ecology as a framework for zoonotic disease risk: Understanding transferability across arenavirus systems

**DOI:** 10.1073/pnas.2625913123

**Published:** 2026-09-25

**Authors:** Gregory C. Milne, Lauren A. Attfield, Joachim Mariën, Lucinda Kirkpatrick, Herwig Leirs, Kate E. Jones, Christl A. Donnelly, David W. Redding

**Affiliations:** ^a^Ecosystem Health Lab, Ecosystem Health Lab, Science Department, Natural History Museum, London SW7 5BD, United Kingdom; ^b^ Environmental Change Institute, School of Geography and the Environment, University of Oxford, Oxford OX1 3QY, United Kingdom; ^c^ Leverhulme Centre for Nature Recovery, School of Geography and the Environment, University of Oxford, Oxford OX1 3QY, United Kingdom; ^d^ Evolutionary Ecology Group, University of Antwerp, Antwerp 2610, Belgium; ^e^ Virus Ecology Group, Institute of Tropical Medicine Antwerp, Antwerp 2000, Belgium; ^f^ School of Environmental and Natural Sciences, Bangor University, Bangor LL57 2DG, Wales; ^g^ Department of Genetics, Evolution and Environment, University College London, London WC1E 6BT, United Kingdom; ^h^ Department of Statistics, University of Oxford, Oxford OX1 3LB, United Kingdom; ^i^ Pandemic Sciences Institute, University of Oxford, Oxford OX3 7DQ, United Kingdom; ^j^ Department of Infectious Disease Epidemiology, Imperial College London, School of Public Health, White City Campus, London W12 0BZ, United Kingdom

**Keywords:** zoonotic disease, rodent-borne disease, population dynamics, arenaviruses, lassa fever

## Abstract

Mechanistic frameworks linking climate to zoonotic disease risk through host ecology remain rare, limiting transferable insights across systems. We develop a Bayesian framework combining an integral projection model of rodent demography with a compartmental disease model. Applied to capture-mark-recapture data from the Natal multimammate mouse (*Mastomys natalensis*; *N* = 20,249 captures, 1994–2023) and serological data on the Lassa virus-related Morogoro arenavirus (*N* = 7,850 tests, 2010–2017), the framework jointly estimates climatic, demographic, and transmission parameters. Rainfall was a dominant driver of rodent recruitment, with seasonal rainfall explaining 52.1% (95% CrI 2.1 to 79.0%) of modeled recruitment variation. In a field-first, we estimate that 79.1% (95% CrI 69.3 to 88.9%) of infected pregnancies result in vertical transmission, identifying this as the primary mechanism maintaining viral persistence between breeding seasons. We assess framework transferability to capture the seasonal dynamics of Lassa fever outbreaks in Nigeria (*N* = 6,469 laboratory-confirmed cases, 2018–2025), substituting model climate inputs without refitting any parameters. Predicted peaks in rodent subadult infections preceded observed outbreaks by 0.92 mo (95% CrI −2.76 to 0.92), with 83% of predicted and observed peaks falling within ±28 d [Pr(Δ ≤ 28d) = 0.83], substantially outperforming seasonal rainfall or rodent demography [Pr(Δ ≤ 28d) ≤ 0.11]. Infected adult peaks lagged observed outbreaks by 0.92 mo [Pr(Δ ≤ 28d) = 0.56], suggesting subadults might primarily be responsible for zoonotic hazard. Outbreak magnitude was not reproduced, likely reflecting human behavioral and surveillance factors beyond the model’s scope. These results illustrate how mechanistic frameworks grounded in reservoir host ecology can yield transferable insights into zoonotic risk, with potential application across other climate-sensitive host–pathogen systems.

## Significance

Climate change is reshaping the distribution and seasonality of zoonotic infectious diseases, yet mechanistic frameworks linking environmental variation to zoonotic spillover risk remain rare. We present a Bayesian framework which integrates climate-driven host population ecology into disease modeling. Applied to the Natal multimammate mouse and its associated arenaviruses, the framework recovers key transmission parameters from field data—including an empirical estimate of vertical transmission probability—and reveals how rainfall-driven recruitment pulses generate periodic infection peaks. By recalibrating the model with Nigerian climate data, predicted rodent infection dynamics reproduce the seasonality of Lassa fever outbreaks, suggesting the framework recovers key ecological insights that generalize across systems; with possible broad applicability to other climate-sensitive zoonotic disease systems.

Climate variability is a well-established driver of human infectious disease risk, shaping the timing, magnitude, and geographic distribution of outbreaks across numerous pathogens (1). However, the mechanisms through which environmental variation translates to disease risk remain poorly understood, limiting our ability to obtain insights that are transferable across systems and regions (2). This challenge is particularly acute for zoonotic diseases, where the pathway from climate variability to human infection runs via animal host ecology; adding biological complexity that purely statistical approaches cannot capture. By contrast, mechanistic frameworks are well established for vector-borne disease systems: Ross-MacDonald-type models linking temperature-dependent mosquito development to pathogen transmission have improved outbreak forecasting (3) and enabled predictions under climate change scenarios (4). Filling the equivalent gap for zoonotic systems is pressing. Rodents host more zoonotic pathogens than any other mammalian order (5), their populations respond rapidly to environmental fluctuation (6), and rodent-borne diseases impose a substantial burden of morbidity and mortality concentrated in low-income settings with limited surveillance capacity (7). Yet mechanistic models linking climate to rodent demography and infection dynamics remain rare (8, 9).

Rodents’ “fast” life-history traits allow rapid demographic responses to resource availability (10), with populations regulated by both density-dependent processes, e.g., competition and predation, and density-independent climatic variation acting directly (e.g., through overwinter survival), and indirectly via resource availability (6, 8, 11–13). These climate-population links have been associated with disease risk across multiple systems, from masting-driven nephropathia epidemica outbreaks in Europe (12), to El Niño-driven hantavirus pulmonary syndrome in the Americas (12). Yet, most existing work is correlational rather than process-based (9, 14–17), with few studies jointly modeling host demography and infection dynamics as interacting processes driven by shared environmental inputs (8). This limits our ability to identify which mechanisms link climate variation to outbreak risk and transfer models across systems. One such rarely quantified mechanism is vertical transmission, the direct passage of pathogen from infected mother to offspring, which may sustain pathogen persistence between breeding seasons even when horizontal transmission is low (18, 19). Quantifying this transmission mechanism requires a process-based framework explicitly linking environmental drivers to host demography and infection dynamics.

Lassa fever (LF) is a climate-sensitive zoonotic disease endemic to West Africa, responsible for an estimated 2.1 to 3.4 million human infections annually (20), most of which remain undiagnosed (21). The Natal multimammate mouse (*Mastomys natalensis*, MN) is the principal reservoir of Lassa mammarenavirus (LASV), the etiological agent of LF, and human cases arise predominantly from direct or indirect contact with infected MN (22); meaning human risk is closely tied to rodent ecology. Strong seasonal patterns in both rodent arenavirus seroprevalence (19, 23, 24) and reported LF cases (25) suggest that climate seasonality drives infection dynamics through the rodent host, yet the mechanisms linking climate, MN demography, and human outbreak timing remain unresolved. This makes LF an ideal but challenging system in which to develop a mechanistic framework: ideal because the ecological pathway from climate to human risk is clear and the disease burden is substantial; challenging because field data on LASV-carrying MN in West Africa are scarce due to biohazard and ethical constraints (26, 27), hindering our ability to build and parameterize inferential frameworks.

A solution lies in Eastern and Southern Africa, where MN has been extensively studied as an agricultural pest and host of several nonpathogenic arenaviruses (19, 23, 28, 29). These arenaviruses share key biological properties with LASV—infections are nonpathogenic in MN (23, 30) and establish as either acute or persistent depending on age at infection (18, 23)—and are well-validated surrogates for the LASV system (23, 31, 32), having informed LASV vaccine development (31, 32) and LASV predictions under anthropogenic change (33). MN populations in Eastern and West Africa show comparable seasonal fluctuations (33), supporting the potential transferability of a framework developed using data-rich East African populations to Lassa-endemic regions. An intensively studied population in Morogoro, Tanzania, with a three-decade capture-mark-recapture (CMR) dataset (29) and paired serological data on the LASV-related Morogoro arenavirus (MORV) (19), provides the empirical foundation for this approach.

Here we develop and apply a mechanistic framework integrating climate-driven host population dynamics with pathogen transmission, parameterized via Bayesian inference on long-term CMR, serological, and climate data from Morogoro. The framework’s core structure rests on embedding a compartmental epidemiological model within an integral projection model (IPM) (34), allowing demographic and epidemiological processes to be driven by shared climate inputs while capturing demographic heterogeneity and explicitly separating horizontal from vertical transmission (*SI Appendix*, Fig. S1) (18, 23). This framework’s three objectives are to 1) quantify the demographic and climatic drivers of MN population dynamics; 2) estimate vertical and horizontal transmission parameters for MORV; and 3) test transferability to observed LF outbreak patterns in Nigeria; serving as proof of concept for whether mechanistic host–pathogen models can yield transferable insights across regions and systems without requiring calibration against human case data.

## Results

### Climate and Demography.

**Fig. 1.**
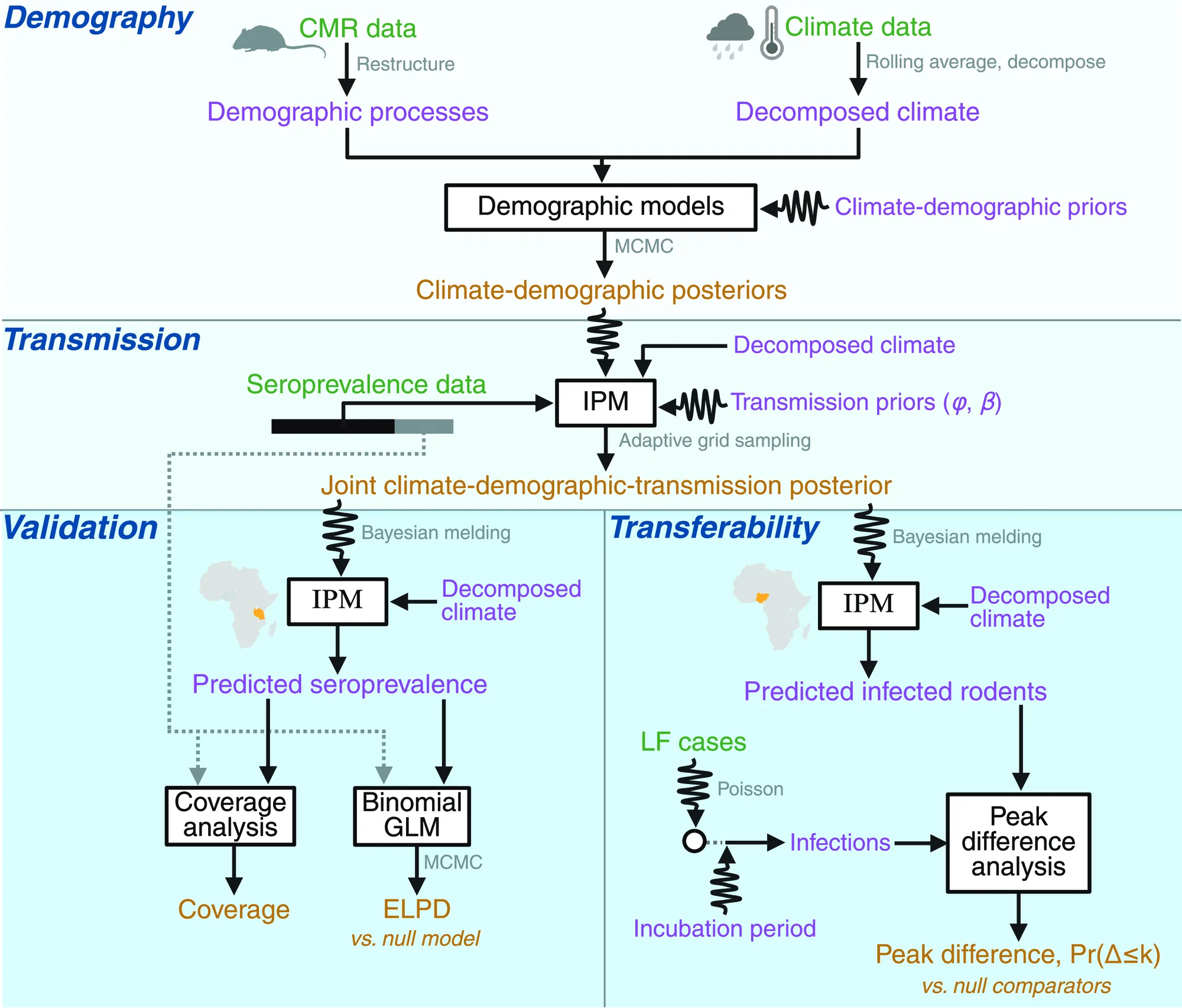
Modeling framework. The framework proceeds in four stages. *Demography:* CMR data on *M. natalensis* (29) and daily climate records (35, 36) are processed to extract demographic processes and decomposed climate components (trend, seasonal, variable). These are used to fit Bayesian demographic GLMs via Markov Chain Monte Carlo (MCMC), yielding climate-demographic posteriors. *Transmission:* these posteriors, together with prior distributions on transmission parameters (*φ*, vertical transmission probability; *β*, horizontal transmission rate), are passed to an IPM (34) via Bayesian adaptive grid sampling to estimate the joint climate-demographic-transmission posterior; seroprevalence data (19) from 2010 to 2016 (black bar) inform estimation and 2017 data (gray bar) are withheld for validation. *Validation:* posterior draws are propagated through the IPM via Bayesian melding (37) and Morogoro seroprevalence estimates validated by 1) proportional overlap between predicted and observed intervals (“coverage”); and 2) regression of predictions against hold-out seroprevalence via a Bayesian binomial GLM, with fit assessed by expected log-predictive density (ELPD) comparison to a null model. *Transferability:* the IPM is recalibrated with Nigerian climate data (from five high-burden states) without refitting parameters, and predicted rodent infection peaks compared to inferred LF infections via a peak difference analysis [Pr(Δ ≤ k)]. Estimates are also compared to null comparators. LF cases (25) are Poisson-sampled to propagate reporting uncertainty and back-projected to infection dates via an incubation period posterior (*SI Appendix*). Text colors: data (green), processes (gray), intermediate products (purple), key outputs (gold). Arrows: direct input/output (straight), sampling (squiggle), external validation data (dotted). Circular nodes represent intermediate sampled data products. The IPM is described mathematically in *Materials and Methods* and depicted in *SI Appendix*, Fig. S1.

In the first framework stage (Fig. 1, *Demography*), Bayesian generalized linear models (GLMs) were fitted to CMR data (*N* = 20,249 captures, 1994–2023) (29) alongside lagged, decomposed climate variables (35, 36), with performance evaluated against climate-free alternatives, to quantify how climate and density-dependent processes shape MN body weight, recruitment, and survival.

**Fig. 2.**
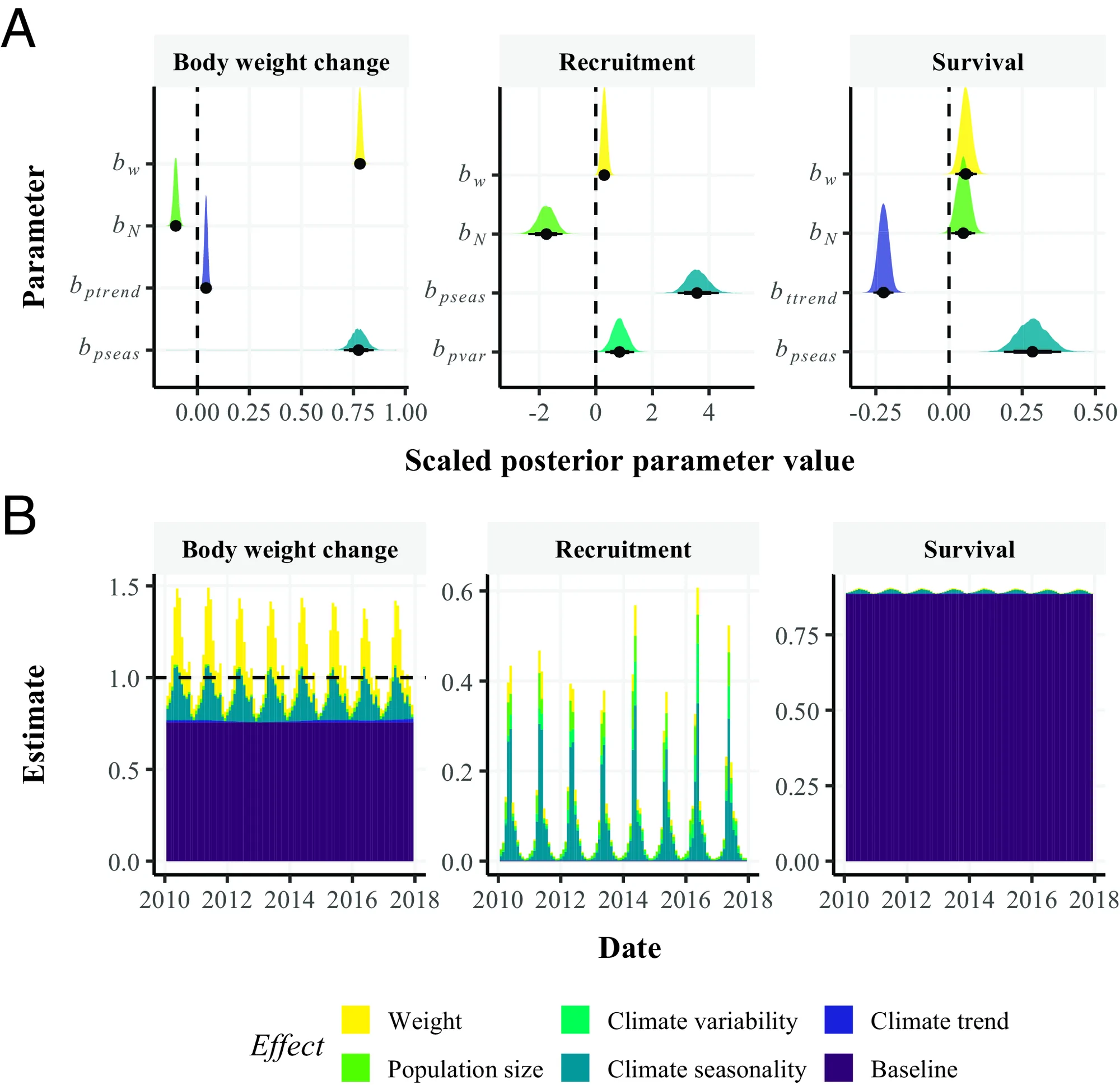
Estimating the demographic and climatic predictors of rodent demography. (*A*) Estimated posterior distributions of unit-scaled coefficients from demographic process models. The density of each posterior distribution is shown alongside a posterior median (point), 80% credible interval (thick horizontal line), and 95% credible interval (thin horizontal line). The vertical dashed line represents no effect. For visual clarity, the uninformative normal prior distributions are not shown in the figure but can be found in *SI Appendix*, Table S1. Notation: $b_{w}$, body weight; $b_{N}$, population size; $b_{\mathrm{ptrend}}$, precipitation trend; $b_{\mathrm{pseas}}$, precipitation seasonality; $b_{\mathrm{pvar}}$, precipitation variability; $b_{\mathrm{ttrend}}$, temperature trend. (*B*) Estimates from *N* = 1,000 posterior predictive simulations of the IPM showing the median contributions of demographic and climatic effects to each demographic process (with colors corresponding to the parameters in panel (*A*). Bars show the time-specific contribution of each covariate to the demographic process, benchmarked against the response that would be predicted if all marginal effects took their minimum value (Baseline). For survival, this baseline value includes the proportion of rodents estimated to be alive despite never being recaptured (*SI Appendix*). The total height of stacked bars is the time-specific predicted response. Units for body weight change are the time-specific predicted weights (grams) made proportional to the mean modeled weight over time (dashed horizontal line), while units for recruitment and survival are probabilities. Demographic process models were fit to decomposed climate data (35, 36) and *M. natalensis* CMR data (29) using a No-U-Turn Sampler algorithm in Stan (38). Posterior predictions were generated using Bayesian melding (37) whereby demographic samples were reweighted based on their consistency with seroprevalence data (19).

Climate was an important predictor of all demographic processes. Models without climate predictors ranked in the lowest 12.5% of candidates by marginal log-likelihood (8/64; *SI Appendix*, Figs. S2–S4), and best-performing models incorporated both demographic predictors (population size and weight) and climatic predictors: precipitation seasonality and trend for body weight change; precipitation seasonality and variability for recruitment; and precipitation seasonality and temperature trend for survival (Fig. 2*A* and *SI Appendix*, Figs. S5–S10 and Table S1).

Rainfall was the dominant climatic driver, with its influence strongest on recruitment (Fig. 2*A* and Table 1); the process most consequential for subsequent infection dynamics, discussed below. Seasonal precipitation explained 52.1% (95% CrI 2.1 to 79.0%) of recruitment variation, with population size accounting for a further 26.4% (95% CrI 1.8 to 55.1%), likely reflecting density-dependent constraints on breeding. Body weight change was jointly driven by previous weight and seasonal precipitation, while survival showed limited dependence on any individual predictor (Table 1 and Fig. 2*B* and *SI Appendix*, Fig. S11). Together, these results establish recruitment as the demographic process most climate-sensitive and most likely to propagate environmental variation into infection dynamics, a hypothesis we evaluate in the next framework stage.

**Table 1.** The drivers of rodent demography

	Body weight change	Recruitment	Survival
Weight	10.82 (1.67 to 27.86)	5.86 (0.74 to 17.02)	0.53 (0.11 to 1.05)
Population size	1.34 (0.10 to 2.40)	26.43 (1.77 to 55.11)	0.37 (0.02 to 1.63)
Climate trend	0.83 (0.01 to 1.68)	NA	0.62 (0.04 to 2.99)
Climate seasonality	11.42 (0.40 to 20.59)	52.12 (2.11 to 78.96)	4.55 (0.20 to 5.34)
Climate variability	NA	11.46 (1.97 to 28.82)	NA

Abbreviations: NA, not applicable (covariate not in model).

Percentage of the variation in modeled demographic process explained by each covariate. Estimates are given as the median posterior predictions with 95% credible intervals (N = 1,000 posterior predictive simulations).

### Transmission Dynamics.

**Fig. 3.**
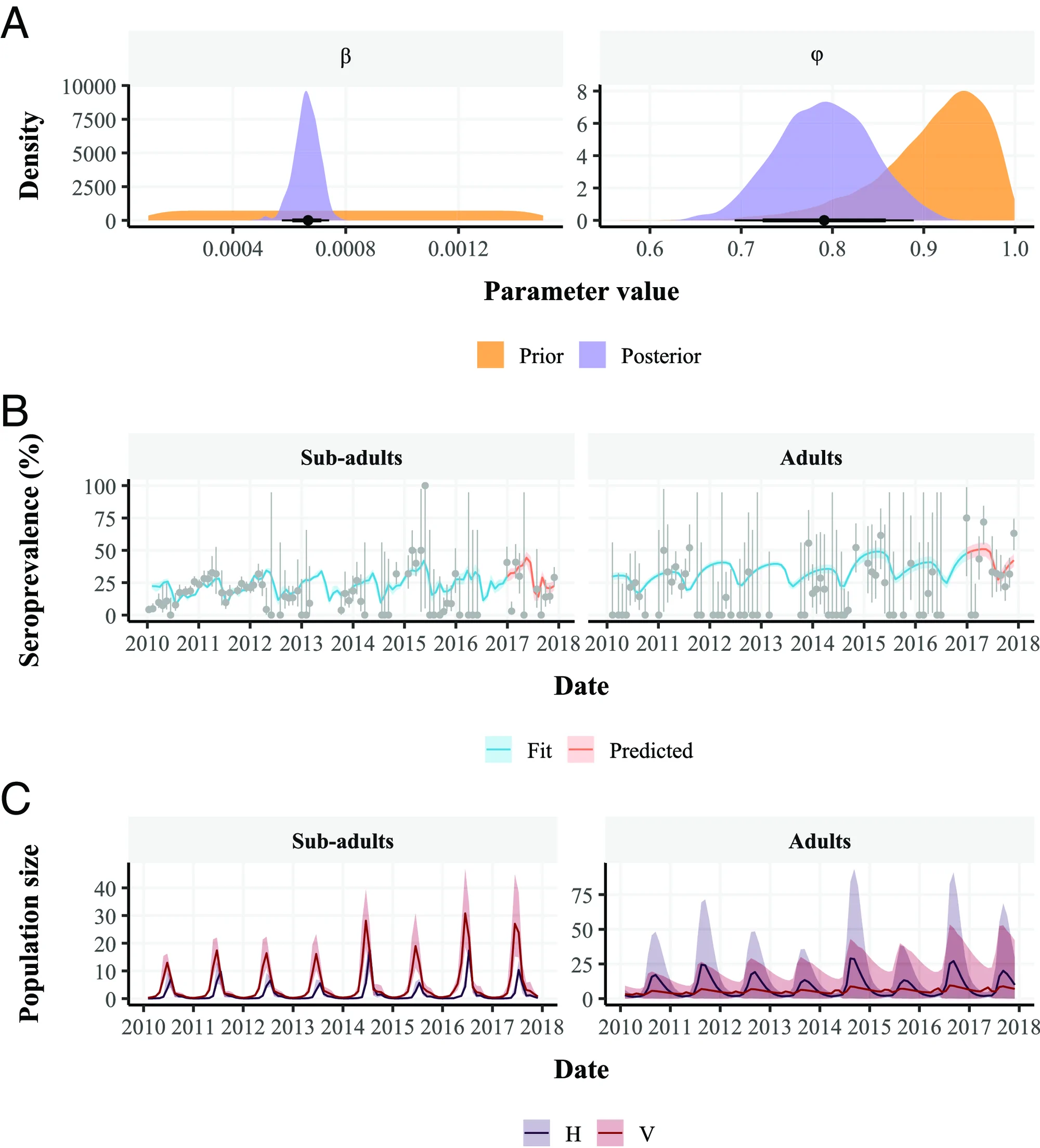
Estimating the drivers of arenavirus transmission. (*A*) Approximate posterior distributions of the horizontal transmission rate (β) and the probability of vertical transmission from an infected pregnancy (φ), with associated prior distributions (uniform and beta, respectively). Units are (28 d)^−1^. Points represent the posterior medians, thick inner lines the 80% credible intervals, and thin outer lines the 95% credible intervals. (*B*) Fit of modeled seroprevalence to observed *M. natalensis* Morogoro arenavirus seroprevalence data for subadults (*N* = 6,904 tests) and adults (*N* = 946 tests) (19). Points and error bars represent observed means and binomial 95% CI for each trapping session, while lines and shaded areas show posterior predictive medians and 95% credible intervals (*N* = 1,000 posterior draws). The IPM was fit to seroprevalence data from 2010–2016 (blue), with 2017 withheld for validation (red). Posterior predictions were generated using Bayesian melding (37) whereby demographic samples were reweighted based on their consistency with seroprevalence data (19). Fit quantification is given in *SI Appendix*, Fig. S12. (*C*) Posterior predictions (medians and 95% credible intervals) from the fitted model of subadult and adult population size by infection compartment: horizontally infected (*H*) and vertically infected (*V*) (*N* = 1,000 posterior draws).

In stage two (Fig. 1, *Transmission*), we estimate transmission by simulating the IPM (*SI Appendix*, Fig. S1) with demographic estimates from stage one while sampling transmission parameters from prior distributions (*SI Appendix*, Table S1), with the resulting joint posterior evaluated against MORV seroprevalence data (*N* = 7,850 tests, 2010–2017) (19). This yields empirical estimates of vertical transmission probability and horizontal transmission rate in any rodent-arenavirus system (*SI Appendix*, Table S1). Vertical transmission probability was estimated at 79.1% per infected pregnancy per 28-d (95% CrI 69.3 to 88.9%) (Fig. 3*A*), indicating the dominant role that mother-to-offspring transmission plays in maintaining viral persistence between breeding seasons.

Seroprevalence predictions were validated in two ways (Fig. 1, *Validation*). First, we assessed the proportion of trapping sessions with overlapping observed and predicted seroprevalences (coverage). Coverage was 88.3% for 2010–2016 training data (106/120; 95% CI 81.4 to 92.9%) and 88.2% for 2017 hold-out data (15/17; 95% CI 65.7 to 96.7) (Fig. 3*B* and *SI Appendix*, Fig. S12), indicating good calibration and no evidence of overfitting (training coverage ≈ hold-out coverage). Second, a Bayesian binomial GLM regressing median predicted seroprevalence against hold-out seroprevalence yielded moderate explanatory power (posterior coefficient = 0.98, 95% CrI 0.62 to 1.34), outperforming an intercept-only null model (*SI Appendix*, *Supplementary Results*); confirming the framework captures seasonal signal rather than average seroprevalence.

We next evaluate the hypothesis that rainfall-driven recruitment is the dominant process linking climate to arenavirus outbreak dynamics. By examining conditions preceding major seroprevalence peaks in 2015 and 2017 (Fig. 3*B*), we find that rainfall anomalies in 2014 and 2016 elevated recruitment probability (Fig. 2*B* and *SI Appendix*, Fig. S13), explaining 17.1% and 17.5% of recruitment variance respectively; substantially exceeding variance explained in smaller outbreak years (8.3 to 11.2%) or the overall model (11.5%; Table 1 and *SI Appendix*, Fig. S11). Mechanistically, elevated recruitment amplified vertical transmission events and, through the subsequent influx of naïve juveniles, horizontal transmission; with the 2016 peak in horizontally infected adults approximately twice that of 2015 (Fig. 3*C* and *SI Appendix*, Figs. S14 and S15). No equivalent shifts were detected in predictors of weight change or survival prior to these outbreaks (*SI Appendix*, Fig. S11), confirming that recruitment, amplified by episodic excess rainfall, is the primary mechanism by which climate drives arenavirus transmission peaks.

### Framework Transferability: LF in Nigeria.

**Fig. 4.**
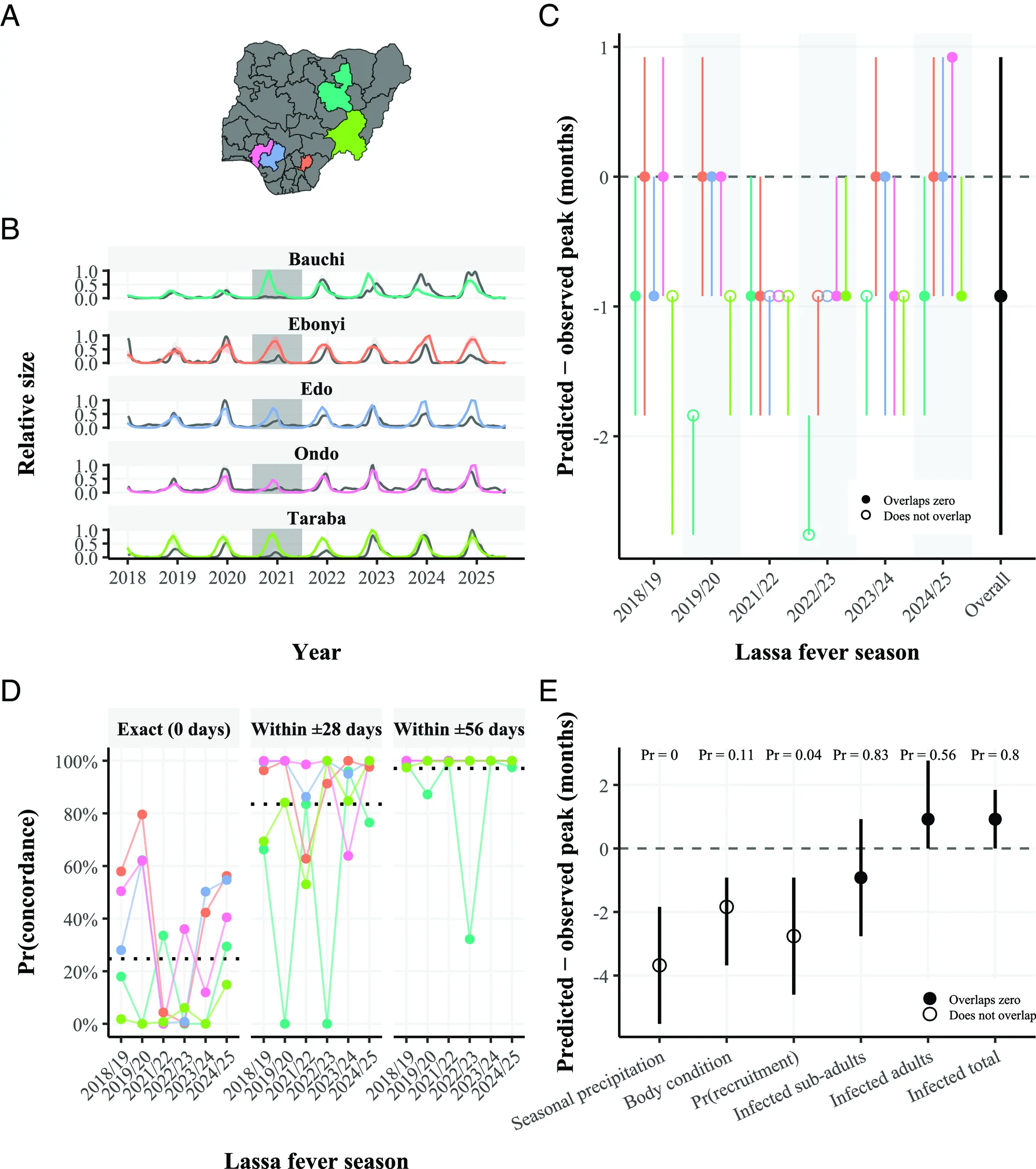
Assessing transferability across arenavirus systems. Nigeria posterior predictive simulations (*N* = 1,000) using the recalibrated model. (*A*) The five included Nigerian states; colors correspond to state names in panel *B*. (*B*) Median (line) and 95% credible interval (CrI; shaded area) of the predicted relative number of infected subadult rodents (color) vs. the relative number of laboratory-confirmed LF cases transformed to infections (gray; *N* = 6,469 cases) (25), shown separately for each state. Uncertainty in reported cases was represented by *N* = 1,000 Poisson-distributed realizations of weekly case counts; estimated infection dates were obtained by convolving each realization with a LF incubation period distribution (sampling *N* = 1,000 random parameter sets from the incubation period posterior; see *SI Appendix*). Predictions and inferred infections were scaled to their respective maxima. Data from the 2020/21 season were omitted due to interruptions in case reporting during the COVID-19 pandemic (dark gray shaded area) (25). (*C*) Differences in peak timing (months) between the predicted number of infected subadult rodents and observed LF outbreaks by state and LF season, with the overall estimate (black) pooled across all states and seasons. The dashed horizontal lines indicate perfect agreement. Peak timing comparisons used *N* = 10,000 bootstrap samples drawn independently from model and observation posterior distributions. (*D*) Probability of concordance between predicted and observed peak timing by state and season across three agreement thresholds: exact agreement (0 d), within ±28 d, and within ±56 d. Dotted horizontal lines show the overall probability of concordance at each threshold, pooled across all states and seasons. (*E*) Peak timing differences summarized across all states and LF seasons for: seasonal precipitation, demographic predictions (body condition and recruitment probability), and infection predictions (subadults, adults, and total). Estimates are shown alongside probability of peak timing concordance within ±28 d. “Infected subadults” is the same metric used in panels *B*–*D*. In panels *C* and *E*, points represent medians and error bars 95% CrIs. LF seasons were defined as July–June to capture the typical December–April outbreak period (25).

In the final stage (Fig. 1, *Transferability*), we recalibrate the IPM with climate data (35, 36) from the five Nigerian states with the highest LF burden (Fig. 4*A*), substituting climate inputs without refitting any parameters, and compare predicted infection dynamics to reported LF cases from 2018–2025 (*N* = 6,469) (25). Since vertically infected subadults are the primary output of rainfall-driven recruitment pulses, we compare observations to predictions for this cohort under the hypothesis that they represent the main zoonotic risk to humans.

Predicted subadult infection dynamics reproduced the seasonal timing of LF outbreaks (Fig. 4*B*), with mean predicted peaks preceding observed peaks by 0.92 mo (95% CrI −2.76 to 0.92; Fig. 4*C*). Across all states and LF seasons, predicted and observed peaks fell within ±28 d of each other in 83% of bootstrap replicates [Pr(Δ ≤ 28 d) = 0.83] and within ±56 d in 97% [Pr(Δ ≤ 56 d) = 0.97; Fig. 4*D*]. By contrast, outbreak timing was not captured by seasonal precipitation or simpler demographic outputs [Pr(Δ ≤ 28 d) ≤ 0.11; Fig. 4*E* and *SI Appendix*, Fig. S16], arguing against coincident seasonality and suggesting the full mechanistic chain of events—climate, demography, transmission—is necessary to capture outbreak timing.

We next evaluated whether the signal of zoonotic hazard is diluted in the adult cohort, as would be expected if subadults represent the primary risk. Peaks in infected adults lagged LF outbreaks by 0.92 mo [95% CrI 0 to 2.76; Pr(Δ ≤ 28 d) = 0.56], with total infections (subadults and adults) lagging by a similar margin [Pr(Δ ≤ 28 d) = 0.80] (Fig. 4*E*). These results suggest that infected subadults may provide an earlier signal of zoonotic hazard than adults or aggregate population metrics and may be the cohort responsible for human zoonotic risk.

Outbreak magnitude, however, was not predicted, consistent with this being governed by human factors outside the framework’s current scope. Predicted peak sizes exceeded observed peaks by 0.02 on average (95% CrI −0.52 to 0.64), with peak sizes falling within ±0.2 relative units in only 43% of bootstrap replicates [Pr(Δ ≤ 0.2) = 0.43; *SI Appendix*, Fig. S17].

## Discussion

By combining long-term ecological data with a mechanistic modeling framework, we demonstrate how climate and demography interact to shape the population and infection dynamics of a major zoonotic reservoir; and show that such frameworks can yield transferable insights across systems and regions without requiring calibration against human case data which are often biased and patchy.

Our models identify precipitation as a key driver across MN demographic processes, consistent with broad evidence linking rainfall to rodent abundance in resource-limited environments (39). The positive relationship between recruitment and episodic rainfall likely reflects increased food availability driving rapid breeding responses (40, 41); a mechanism that becomes increasingly ecologically consequential as climate change alters the frequency and intensity of rainfall anomalies in semiarid systems like Morogoro (42). The strong El Niño event of 2015–2016, which brought exceptional rainfall to East Africa (43), coincided with the predicted recruitment spike of 2016 (Fig. 2*B*). The potential for such events to become more frequent or severe underscores the value of mechanistic frameworks that can translate projected climate inputs into predictions of reservoir population and infection dynamics.

While two-thirds of the variation in recruitment was explained through precipitation it was also substantially constrained by population size; reflecting density-dependent regulation of breeding (6, 44). Population size also had negative effects on weight gain—likely reflecting competition—but a positive effect on survival, possibly due to dilution of per-capita predation risk in larger populations (45). Weight was an important predictor of survival and reproduction, consistent with evidence that body condition buffers starvation risk and enhances fecundity (6, 8, 46). These results indicate that climate drivers act within the ecological context of the system.

Our framework builds on previous foundational work (19) to provide empirical estimates of arenavirus vertical transmission probability. Our estimate that 79.1% (95% CrI 69.3 to 88.9%) of infected pregnancies transmit MORV to offspring is broadly consistent with laboratory estimates for LASV (99.3%) and MORV (100%) (18, 23), with discrepancies plausibly attributable to genetic and immunity differences between wild and laboratory rodents (47). Our result agrees with previous studies identifying vertical transmission as a dominant mechanism sustaining viral persistence between breeding seasons, when horizontal transmission rates are low due to reduced encounter rates between infectious and susceptible individuals (18, 19, 23, 24). This has direct implications for control strategies: Fertility management could simultaneously reduce the number of new recruits and the number of vertically infected offspring, whereas untargeted culling is unlikely to be effective since many juveniles are already infected (19, 44).

Vertical transmission is likely underappreciated as a mechanism of interseasonal pathogen persistence across zoonotic systems more broadly. Relevant examples span rodent, bat, and vector-borne systems: Tick-borne encephalitis virus has been detected in up to 90% of offspring from naturally infected red voles (48); vertical transmission is suspected to contribute to the enzootic maintenance of Ebola virus in bats (49); and vertical transmission in *Aedes* mosquitoes contributes to Rift Valley fever persistence (50), with emerging evidence of vertical transmission in ruminant hosts (51). Our framework could in principle quantify this route across such systems, provided three data requirements are met: longitudinal serology with age or reproductive-stage information; demographic data at sufficient temporal resolution to estimate demographic processes; and a host in which vertically and horizontally infected individuals are distinguishable via antibody or shedding dynamics.

The LF transferability test illustrates a key strength of the framework: calibrating models on a data-rich system yields ecological insights on a data-sparse endemic setting (52). Correspondence between predicted infected subadult rodents and LF outbreak timing—but not climatic or demographic drivers alone—argues against coincident seasonality and identifies rodent infection dynamics as the proximate determinant of zoonotic hazard (22, 53). Concordant results despite phylogeographic differences between LASV-carrying MN in Nigeria and MORV-carrying MN in Tanzania (28) may reflect their comparable demographic responses to climatic cues (33).

Our finding that the framework, without being fit to human case data, successfully recovers approximate outbreak timing but not magnitude suggests that rodent infection dynamics set the temporal window of zoonotic hazard, while human factors determine how much of that hazard is realized as reported cases. Our result that subadult infection peaks precede human outbreaks, while adult infection peaks do not, may be due to the prolonged shedding of chronically infectious subadults compared with transiently infectious adults (18, 19, 23, 24), or may be a limitation of the model’s 4-wk time step.

Key limitations of our study bear acknowledgment. Agricultural practices influencing MN movement and abundance (40) were excluded due to data unavailability; their future inclusion could inform finer-scale models linking rodent ecology to human exposure (54). The assumption of a homogenously mixed population is consistent with MN’s nonterritorial social structure (55) but may not capture fine-scale spatial heterogeneity in contact rates (56, 57).

Future studies wishing to build mechanistic forecasting tools for LF could adapt our framework by adding an interface to human infection dynamics while estimating rodent–human contacts using LF case data (58). Such an approach would need to further consider the differences between the Nigerian and Tanzanian systems—in factors such as host specificity (18, 59), virus species (23, 31, 32), habitat use (58), and rodent contact rates (56, 57)—otherwise risk conflating rodent–human contact rates with other confounding variables. Ultimately, it is likely that data unavailability (52) will require some assumption of congruence to be made between these disparate rodent systems.

In conclusion, we present a mechanistic framework linking climate, host demography, and pathogen transmission; parameterized from field data and transferable across systems without requiring human case data for calibration. The framework offers a template for bridging the methodological gap between well-developed vector-borne disease models and the far larger set of zoonotic systems where equivalent approaches remain rare, with potential to inform outbreak preparedness tools under a changing climate.

## Materials and Methods

### Data.

We extracted publicly available CMR data on MN from a 29-y study in Morogoro, Tanzania (29). Trapping occurred every 28 d over three consecutive nights, yielding 377 sessions over 1,083 trap days (29). We excluded records with missing or erroneous values, classified individuals as subadults or adults based on reproductive condition (29), and calculated the Minimum Number Alive as an index of relative abundance (60). Analyses were restricted to females (*N* = 8,628 individuals, 20,249 captures, 1994–2023), under the assumption that male availability was not limiting for reproduction.

We obtained MORV seroprevalence data collected 2007–2017 using indirect immunofluorescence (19), excluding observations from 2007–2009 due to quality issues (J. Mariën, pers. comm.) and records with missing/ambiguous serostatus. Seroprevalence was estimated per session for subadults and adults with 95% binomial CI. As sex was not a significant predictor of exposure (multivariable GLM, *P* = 0.33), data were pooled across sexes, yielding 3,598 individuals and 7,850 tests across 79 sessions.

Daily precipitation and temperature was extracted at the trapping site (35, 36) and used to calculate 28-d rolling averages. We applied seasonal-trend decomposition via LOESS (61) to extract trend, seasonal, and residual (hereafter “variable”) components; window parameters and data cleaning steps are described in *SI Appendix*.

### Demographic Models.

We developed Bayesian hierarchical GLMs in Stan (38) for body weight change (hereafter “growth”), recruitment, and survival, each modeled as a function of individual body weight $X_{w}$, time-specific population size $X_{N}\left(t\right)$ [allowing for density-dependent effects like competition and predation (6, 44)], and decomposed climate covariates, $c_{i}$. For each process, the linear predictor was

[1] $$g\left(\mu \right)\;=\;a\;+\;b_{w}X_{w}\;+\;b_{N}X_{N}\left(t\right)\;+\;\sum_{i}\sum_{k}b_{i}\omega_{k}c_{i}(t\;-\;L_{i,k}),$$

where $g\left(\mu \right)$ is the link function (identity for growth, logit for recruitment, and survival), a is an intercept, $b_{w}$ and $b_{N}$ are coefficients on body weight and population size, $b_{i}$ is a coefficient on climate component i, $L_{i,k}$ is the k th temperature/precipitation lag (0 to 168 d in 28-d intervals) corresponding to climate covariate i, and $\omega_{k}$ is the associated lag weight. Estimating $\omega_{k}$ allows past climates to contribute to current demographic rates in proportion to their explanatory power.

Growth was estimated from paired captures of nonpregnant, nonlactating females using a heteroskedastic normal GLM. Recruitment was defined as individuals captured as nonpregnant, nonlactating at time t and pregnant or lactating at t+1, or lactating at t+2 (62). Survival was not directly observable; recapture probability was estimated from individual recapture histories and adjusted to give survival (*SI Appendix*). Covariates were unit-scaled with weakly informative zero-centered prior distributions (*SI Appendix*, Table S1). For each process, 64 candidate models were fitted representing all combinations of six climate predictors; the top-ranked model by marginal log-likelihood (63) was used for inference. MCMC fitting details, convergence diagnostics, and posterior predictive checks are described in *SI Appendix*.

### Population and Infection Model.

The fitted demographic GLMs Eq. Eq. **1** were used to construct an IPM (34) of rodent population and pathogen dynamics (https://github.com/gcmilne/lassa-popdyn and *SI Appendix*, Fig. S1), tracking the body weight distribution in discrete 28-d time steps:

[2] $$n\left(w^{\prime },t\;+\;1\right)\;=\;\int \left[P(w\prime ,w,t)\;+\;F(w\prime ,w,t)\right]n(w,t)dw,$$

where $n\left(w,t\right)$ is the density of individuals of weight w at time t, P(w′,w,t) is the weight-dependent probability of survival and growth to weight w′ at t+1, and F(w′,w,t) is the density of recruited individuals at weight w′ from parents of weight w.

A compartmental model was embedded within the IPM with compartments susceptible (S), horizontally infected (H), vertically infected (V), and recovered (R) updated by

$$S_{t\;+\;1}\;=\;\left(1\;-\;\lambda_{t}\right)p_{S,t}S_{t}\;+\;\eta p_{S,t}R_{t}\;+\;B_{S,t},$$

$$H_{t\;+\;1}\;=\;(1\;-\;\gamma )p_{S,t}H_{t}\;+\;\lambda_{t}p_{S,t}S_{t},$$

$$V_{t\;+\;1}\;=\;p_{S,t}V_{t}\;+\;B_{V,t},$$

[3] $$R_{t\;+\;1}\;=\;\left(1\;-\;\eta \right)p_{S,t}R_{t}\;+\;\gamma p_{S,t}H_{t}\;+\;B_{R,t},$$

where $p_{S,t}$ is the survival probability from Eq. **1**; $\lambda_{t}$ is the horizontal force of infection; and $B_{S,t}$, $B_{V,t}$, and $B_{R,t}$ are births entering each compartment. Births from H and V mothers produce vertically infected offspring with vertical transmission probability φ and susceptible offspring with probability 1-φ, while recovered mothers produce offspring with transient antibodies that wane at rate η (19, 64). Horizontally infected individuals recover at rate γ, while vertical infection is lifelong (18, 23, 24). Horizontal transmission is implemented by estimating a density-dependent horizontal transmission rate $\beta \left(t\right)$ [consistent with MN’s nonterritoriality (55)] and then approximating the effective per-time-step force of infection $\lambda_{t}$ (*SI Appendix*). Within the IPM, the compartments and births become densities across body weights and survival probability becomes weight-specific (*SI Appendix*).

### Transmission Estimation and Posterior Prediction.

We approximated the joint posterior distribution of (φ,β) using a Bayesian adaptive grid search alongside MORV seroprevalence data (withholding 2017 for validation; Fig. 1, *Transmission*) (19). The algorithm estimated the posterior probability surface $PD_{i}\left(\varphi ,\beta \right)$ across climate-demographic posterior draws (i∈{1,2,..,1000}) given prior distributions (*SI Appendix*, Table S1) and seroprevalence predictions [[*N*(*t*) − *S*(*t*)]/*N*(*t*), reflecting life-long seropositivity (23)]. An initial coarse grid search was refined iteratively to yield a smoother $PD_{i}\left(\varphi ,\beta \right)$ used for posterior predictions.

Posterior predictions were generated using Bayesian melding (Fig. 1, *Validation* and *Transferability*) (37), sampling from climate-demographic posteriors and transmission posteriors while ensuring climate-demographic draws consistent with the seroprevalence data receive higher weight. Briefly, for i∈{1,2,..,1000} iterations, demographic posterior draw i was resampled with probability proportional to its marginal posterior mass $PD_{i}\left(\varphi ,\beta \right)$. Conditional on draw i, a (φ,β) pair was sampled from $PD_{i}\left(\varphi ,\beta \right)$ and the IPM simulated. Outputs were summarized as medians and 95% CrIs (0.5, 0.025, and 0.975 quantiles).

### Validation.

To validate seroprevalence predictions (Fig. 1, *Validation*), we first estimated the proportion of observation 95% CrIs overlapping predicted 95% CrIs (hereafter “coverage”). We compared coverage for training data (2010–2016) and hold-out data (2017), assessing overfitting (training coverage >> hold-out coverage) versus out-of-sample predictive skill (hold-out coverage ≈ training coverage) (*SI Appendix*, Fig. S12). Second, we regressed median predicted seroprevalence ${\hat{\theta }}_{\mathrm{gt}}$ against hold-out observations using a Bayesian binomial GLM with identity link, fitted via MCMC in *brms* (65):

[4] $$M_{\mathrm{gt}}∼\text{Bin}(n_{\mathrm{gt}},a\;+\;b{\hat{\theta }}_{\mathrm{gt}}),$$

where $M_{\mathrm{gt}}$ is number of seropositive rodents for group g (subadults and adults) at time t, and $n_{\mathrm{gt}}$ is number of rodents tested. A posterior coefficient b of 1 indicates 1:1 prediction:observation concordance. We compared this model to an intercept-only null model by approximate leave-one-out cross-validation on expected log-predictive densities (65).

### Framework Transferability.

We assessed framework transferability to capture LF dynamics (Fig. 1, *Transferability*), substituting Morogoro’s for Nigeria’s climate (35, 36) extracted at the centroid of the five highest-burden states (Fig. 4*A*), without refitting any parameters. We compared *N* = 1,000 posterior predictions of infected subadults (H+V) to laboratory-confirmed LF cases (2018–2025, *N* = 6,469) (25, 66), using data ≥2018 due to changes in testing capacity (67) and excluding 2020/21 due to reporting interruptions caused by COVID-19 (25).

We propagated uncertainty in true case burden by generating Poisson-distributed realizations of weekly reported cases:

[5] $${\hat{Y}}_{s,j}\left(d\right)∼\mathrm{Pois}\left(\lambda \;=\;Y_{s}\left(d\right)\right),$$

where $Y_{s}\left(d\right)$ is the reported count on day d in state s. To obtain an estimated LASV infection time series $I_{s,j}\left(d\right)$, we back-projected each realization ${\hat{Y}}_{s,j}\left(d\right)$ by drawing, for each j∈{1,2,...,1000}, a single parameter set $\theta_{j}$ from a LF incubation period posterior distribution (*SI Appendix*), defining a lognormal distribution $p_{I}\left(∙;\theta_{j}\right)$:

[6] $$I_{s,j}\left(d\right)\;=\;\sum_{i=1}^{30}{\hat{Y}}_{s,j}\left(d\;+\;i\right)p_{I}(i;\theta_{j}),$$

where $p_{I}\left(i;\theta_{j}\right)$ is the probability that an infection occurring on day d becomes symptomatic i days later. Each $I_{s,j}\left(d\right)$ was aggregated into 28-d windows and scaled to its maximum to yield a relative metric comparable to scaled model predictions.

We extracted the window in which predictions and observations peaked for each iteration/realization, state and LF season, then drew *N* = 10,000 independent bootstrap pairs from these distributions and computed differences as Δ = model—observed peak window. Differences were summarized as posterior medians and 95% CrIs and concordance was quantified as Pr(|Δ| ≤ k) for windows k∈{0,1,2} (exact agreement, ±28 d, ±56 d).

To evaluate the added value of the full mechanistic chain, model peak timing was compared against three null comparators: seasonal precipitation, recruitment probability, and adult body condition; using Pr(|Δ| ≤ 1) as the primary concordance metric. Peak timing was additionally compared separately for infected adults to assess age-class specificity of the outbreak signal.

## Supplementary Material

Appendix 01 (PDF)

## Data Availability

Morogoro arenavirus seroprevalence data are available as grouped data in ref. 19; the individual-level data can be found in a Dryad repository (68). Capture-mark-recapture data are available as previously published open-source data from ref. 29. Daily climate data are available from refs. 35 and 36. The code used for the analyses performed in this paper can be found in Github (69).

## References

[1] S. Altizer, R. S. Ostfeld, P. T. J. Johnson, S. Kutz, C. D. Harvell, Climate change and infectious diseases: From evidence to a predictive framework. Science **341**, 514–519 (2013). 10.1126/science.1239401 23908230

[2] A. Pfenning-Butterworth , Interconnecting global threats: Climate change, biodiversity loss, and infectious diseases. Lancet Planet. Health **8**, e270–e283 (2024). 10.1016/S2542-5196(24)00021-4 PMC11090248 38580428

[3] J. M. Caldwell , Climate predicts geographic and temporal variation in mosquito-borne disease dynamics on two continents. Nat. Commun. **12**, 1233 (2021). 10.1038/s41467-021-21496-7 PMC7902664 33623008

[4] S. J. Ryan, C. J. Carlson, E. A. Mordecai, L. R. Johnson, Global expansion and redistribution of Aedes-borne virus transmission risk with climate change. PLoS Negl. Trop. Dis. **13**, e0007213 (2019). 10.1371/journal.pntd.0007213 PMC6438455 30921321

[5] B. A. Han, A. M. Kramer, J. M. Drake, Global patterns of zoonotic disease in mammals. Trends Parasitol. **32**, 565–577 (2016). 10.1016/j.pt.2016.04.007 PMC4921293 27316904

[6] H. P. Andreassen , Population cycles and outbreaks of small rodents: Ten essential questions we still need to solve. Oecologia **195**, 601–622 (2020). 10.1007/s00442-020-04810-w PMC7940343 33369695

[7] F. Keesing, R. S. Ostfeld, Emerging patterns in rodent-borne zoonotic diseases. Science **385**, 1305–1310 (2024). 10.1126/science.adq7993 39298587

[8] E. M. Soininen, M. Neby, Small rodent population cycles and plants– After 70 years, where do we go? Biol. Rev. **99**, 265–294 (2024). 10.1111/brv.13021 37827522

[9] S. Carver , Toward a mechanistic understanding of environmentally forced zoonotic disease emergence: Sin Nombre Hantavirus. BioScience **65**, 651–666 (2015). 10.1093/biosci/biv047 PMC4776718 26955081

[10] B. A. Han, J. P. Schmidt, S. E. Bowden, J. M. Drake, Rodent reservoirs of future zoonotic diseases. Proc. Natl. Acad. Sci. U.S.A. **112**, 7039–7044 (2015). 10.1073/pnas.1501598112 PMC4460448 26038558

[11] X. Wan , Broad-scale climate variation drives the dynamics of animal populations: A global multi-taxa analysis. Biol. Rev. **97**, 2174–2194 (2022). 10.1111/brv.12888 35942895

[12] H. Tian, N. C. Stenseth, The ecological dynamics of hantavirus diseases: From environmental variability to disease prevention largely based on data from China. PLoS Negl. Trop. Dis. **13**, e0006901 (2019). 10.1371/journal.pntd.0006901 PMC6383869 30789905

[13] F. Barraquand , Moving forward in circles: Challenges and opportunities in modelling population cycles. Ecol. Lett. **20**, 1074–1092 (2017). 10.1111/ele.12789 28633194

[14] H. Tian , Interannual cycles of Hantaan virus outbreaks at the human-animal interface in Central China are controlled by temperature and rainfall. Proc. Natl. Acad. Sci. U.S.A. **114**, 8041–8046 (2017). 10.1073/pnas.1701777114 PMC5544290 28696305

[15] G. Marini, D. Arnoldi, A. Rizzoli, V. Tagliapietra, Estimating rodent population abundance using early climatic predictors. Eur. J. Wildl. Res. **69**, 1–7 (2023).

[16] N. C. Stenseth , Plague dynamics are driven by climate variation. Proc. Natl. Acad. Sci. U.S.A. **103**, 13110–13115 (2006). 10.1073/pnas.0602447103 PMC1559761 16924109

[17] A. Swart , Modelling human Puumala hantavirus infection in relation to bank vole abundance and masting intensity in the Netherlands. Infect. Ecol. Epidemiol. **7**, 1287986 (2017). 10.1080/20008686.2017.1287986 PMC5443058 28567209

[18] C. Hoffmann , Lassa virus persistence with high viral titers following experimental infection in its natural reservoir host. *Mastomys natalensis*. Nat. Commun. **1**, 9319 (2024). 10.1038/s41467-024-53616-4 PMC11522386 39472431

[19] J. Mariën , Density dependence and persistence of Morogoro arenavirus transmission in a fluctuating population of its reservoir host. J. Anim. Ecol. **89**, 506–518 (2020). 10.1111/1365-2656.13107 31545505

[20] D. R. M. Smith , Health and economic impacts of Lassa vaccination campaigns in West Africa. Nat. Med. **12**, 1–10 (2024). 10.1038/s41591-024-03232-y PMC11645265 39198710

[21] J. Mariën , Serosurveillance identifies an endemic hotspot of Lassa fever in Faranah. Upper Guinea. J. Infect. Dis. **232**, e830–e838 (2025). 10.1093/infdis/jiaf308 PMC12614985 40509999

[22] K. J. Siddle , Genomic analysis of Lassa virus during an increase in cases in Nigeria in 2018. N. Engl. J. Med. **379**, 1745–1753 (2018). 10.1056/NEJMoa1804498 PMC6181183 30332564

[23] B. Borremans , Shedding dynamics of Morogoro virus, an African arenavirus closely related to Lassa virus, in its natural reservoir host *Mastomys natalensis*. Sci. Rep. **5**, 1–8 (2015). 10.1038/srep10445 PMC4448520 26022445

[24] J. Mariën , Arenavirus dynamics in experimentally and naturally infected rodents. EcoHealth **14**, 463–473 (2017). 10.1007/s10393-017-1256-7 28616660

[25] Nigeria Centre for Disease Control and Prevention, An update of Lassa fever outbreak in Nigeria (2025). https://ncdc.gov.ng/diseases/sitreps/?cat=5&name=An%20update%20of%20Lassa%20fever%20outbreak%20in%20Nigeria update of Lassa fever outbreak in Nigeria.

[26] J. Mariën, F. Kourouma, N. F. Magassouba, H. Leirs, E. Fichet-Calvet, Movement patterns of small rodents in Lassa fever-endemic villages in Guinea. EcoHealth **15**, 348–359 (2018). 10.1007/s10393-018-1331-8 29572697

[27] A. S. Adesina , Circulation of Lassa virus across the endemic Edo-Ondo axis, Nigeria, with cross-species transmission between multimammate mice. Emerg. Microbes Infect. **12**, 2219350 (2023). 10.1080/22221751.2023.2219350 PMC10251791 37288752

[28] A. Hánová , Historical demography and climatic niches of the Natal multimammate mouse (Mastomys natalensis) in the Zambezian region. Mamm. Biol. **103**, 239–251 (2023).

[29] H. Leirs , Twenty-nine years of continuous monthly capture-mark-recapture data of multimammate mice (*Mastomys natalensis*) in Morogoro, Tanzania. Sci. Data **10**, 1–8 (2023). 10.1038/s41597-023-02700-3 PMC10640561 37952006

[30] J. Mariën , No measurable adverse effects of Lassa, Morogoro and Gairo arenaviruses on their rodent reservoir host in natural conditions. Parasites Vectors **10**, 210 (2017). 10.1186/s13071-017-2146-0 PMC5408478 28449693

[31] X. Carnec , A vaccine platform against arenaviruses based on a recombinant hyperattenuated mopeia virus expressing heterologous glycoproteins. J. Virol. **92**, 2230–2247 (2018). 10.1128/JVI.02230-17 PMC5974477 29593043

[32] I. S. Lukashevich , A live attenuated vaccine for Lassa fever made by reassortment of Lassa and Mopeia viruses. J. Virol. **79**, 13934–13942 (2005). 10.1128/JVI.79.22.13934-13942.2005 PMC1280243 16254329

[33] R. S. John, H. O. Fatoyinbo, D. T. S. Hayman, Modelling Lassa virus dynamics in West African Mastomys natalensis and the impact of human activities. J. R. Soc. Interface **21**, 20240106 (2024). 10.1098/rsif.2024.0106 PMC11267396 39045680

[34] S. P. Ellner, M. Rees, Integral projection models for species with complex demography. Am. Nat. **167**, 410–428 (2006). 10.1086/499438 16673349

[35] National Oceanic and Atmospheric Administration, Climate Prediction Centre global daily temperature (2025). https://psl.noaa.gov/data/gridded/data.cpc.globaltemp.html.

[36] National Oceanic and Atmospheric Administration, Climate Prediction Centre global daily precipitation (2025). https://psl.noaa.gov/data/gridded/data.cpc.globalprecip.html.

[37] L. Alkema, A. E. Raftery, S. J. Clark, Probabilistic projections of HIV prevalence using Bayesian melding. Ann. Appl. Statist. **1**, 229–248 (2007).

[38] B. Carpenter , Stan: A probabilistic programming language. J. Stat. Softw. **76**, 1–32 (2017). 10.18637/jss.v076.i01 PMC9788645 36568334

[39] P. D. Ventura-Rojas, A. González-Romero, C. E. Moreno, V. J. Sosa, Effect of rainfall, temperature and climate change on the ecology of the rodents of arid zones: A review. Mamm. Rev. **55**, e12372 (2025).

[40] E. Fichet-Calvet , Fluctuation of abundance and Lassa virus prevalence in *Mastomys natalensis* in Guinea, West Africa. Vector Borne Zoonotic Dis. **7**, 119–128 (2007). 10.1089/vbz.2006.0520 17627428

[41] E. C. M. Mlyashimbi , Relationships between seasonal changes in diet of Multimammate rat (Mastomys natalensis) and its breeding patterns in semi-arid areas in Tanzania. Cogent Food Agric. **4**, 1507509 (2018).

[42] D. S. Magang, M. A. Ojara, L. Yunsheng, P. H. King’uza, Future climate projection across Tanzania under CMIP6 with high-resolution regional climate model. Sci. Rep. **14**, 12741 (2024). 10.1038/s41598-024-63495-w PMC11148196 38830967

[43] S. R. Kolusu , The El Niño event of 2015–2016: Climate anomalies and their impact on groundwater resources in East and Southern Africa. Hydrol. Earth Syst. Sci. **23**, 1751–1762 (2019).

[44] J. Mariën , Rodent control strategies and Lassa virus: Some unexpected effects in Guinea, West Africa. Emerg. Microbes Infect. **13**, 2341141 (2024). 10.1080/22221751.2024.2341141 PMC11034454 38597241

[45] J. Lehtonen, K. Jaatinen, Safety in numbers: The dilution effect and other drivers of group life in the face of danger. Behav. Ecol. Sociobiol. **70**, 449–458 (2016).

[46] F. S. Dobson, M. K. Oli, “Chapter 8: Fast and slow life histories of rodents” in Rodent Societies: An Ecological and Evolutionary Perspective, J. O. Wolff, P. W. Sherman, Eds. (University of Chicago Press, 2008).

[47] S. Abolins , The comparative immunology of wild and laboratory mice. *Mus musculus domesticus*. Nat. Commun. **8**, 14811 (2017). 10.1038/ncomms14811 PMC5418598 28466840

[48] V. N. Bakhvalova, O. F. Potapova, V. V. Panov, O. V. Morozova, Vertical transmission of tick-borne encephalitis virus between generations of adapted reservoir small rodents. Virus Res. **140**, 172–178 (2009). 10.1016/j.virusres.2008.12.001 19111585

[49] S. A. Riesle-Sbarbaro , Selective replication and vertical transmission of Ebola virus in experimentally infected Angolan free-tailed bats. Nat. Commun. **15**, 925 (2024). 10.1038/s41467-024-45231-0 PMC10830451 38297087

[50] N. Chitnis, J. M. Hyman, C. A. Manore, Modelling vertical transmission in vector-borne diseases with applications to Rift Valley fever. J. Biol. Dyn. **7**, 11–40 (2013). 10.1080/17513758.2012.733427 PMC4260360 23098257

[51] B. Tinto , Rift Valley fever in West Africa: A zoonotic disease with multiple socio-economic consequences. One Health **17**, 100583 (2023). 10.1016/j.onehlt.2023.100583 PMC10474305 37664171

[52] D. Simons, L. A. Attfield, K. E. Jones, D. Watson-Jones, R. Kock, Rodent trapping studies as an overlooked information source for understanding endemic and novel zoonotic spillover. PLoS Negl. Trop. Dis. **17**, e0010772 (2023). 10.1371/journal.pntd.0010772 PMC9894545 36689474

[53] J. Q. McKendrick, W. S. D. Tennant, M. J. Tildesley, Modelling seasonality of Lassa fever incidences and vector dynamics in Nigeria. PLoS Negl. Trop. Dis. **17**, e0011543 (2023). 10.1371/journal.pntd.0011543 PMC10681316 37956170

[54] R. K. McCormack, L. J. S. Allen, Multi-patch deterministic and stochastic models for wildlife diseases. J. Biol. Dyn. **1**, 63–85 (2007). 10.1080/17513750601032711 22880613

[55] B. Borremans , Happily together forever: Temporal variation in spatial patterns and complete lack of territoriality in a promiscuous rodent. Popul. Ecol. **56**, 109–118 (2014).

[56] G. F. Albery , Density-dependent network structuring within and across wild animal systems. Nat. Ecol. Evol. **9**, 2002–2013 (2025). 10.1038/s41559-025-02843-z 40908382

[57] L. Kirkpatrick, J. Mariën, C. Sabuni, B. Mwamundela, H. Leirs, Partitioning social and spatial drivers of infection risk. Ecol. Evol. **15**, e72367 (2025). 10.1002/ece3.72367 PMC12580238 41201022

[58] D. W. Redding , Geographical drivers and climate-linked dynamics of Lassa fever in Nigeria. Nat. Commun. **12**, 1–10 (2021). 10.1038/s41467-021-25910-y PMC8486829 34599162

[59] S. Gryseels , When viruses don’t go viral: The importance of host phylogeographic structure in the spatial spread of arenaviruses. PLoS Pathog. **13**, e1006073 (2017). 10.1371/journal.ppat.1006073 PMC5226678 28076397

[60] M. E. Graipel, M. I. M. Hernández, C. Salvador, Evaluation of abundance indexes in open population studies: A comparison in populations of small mammals in southern Brazil. Braz. J. Biol. **74**, 553–559 (2014). 10.1590/bjb.2014.0077 25296202

[61] R. B. Cleveland, W. S. Cleveland, J. E. McRae, I. Terpenning, STL: A seasonal-trend decomposition procedure based on loess. J. Off. Stat. **6**, 3–73 (1990).

[62] J. Meester, Early post-natal development of multi-mammate mice Rattus (Mastomys) natalensis (A. Smith). Ann. Transvaal Museum **24**, 35–52 (1960).

[63] Q. F. Gronau, H. Singmann, E. J. Wagenmakers, Bridgesampling: An R package for estimating normalizing constants. J. Stat. Softw. **92**, 1–29 (2020).

[64] E. Fichet-Calvet, B. Becker-Ziaja, L. Koivogui, S. Günther, Lassa serology in natural populations of rodents and horizontal transmission. Vector Borne Zoonotic Dis. **14**, 665–674 (2014). 10.1089/vbz.2013.1484 PMC4170823 25229705

[65] P. C. Bürkner, brms: An R package for Bayesian multilevel models using Stan. J. Stat. Softw. **80**, 1–28 (2017).

[66] A. Trebski, Lassa fever NCDC reports sourcing pipeline (2025). https://github.com/BioDivHealth/LF_Nigeria_Reports (2025).

[67] E. A. Ilori , Increase in Lassa fever cases in Nigeria, January-March 2018. Emerg. Infect. Dis. **25**, 1026 (2019). 10.3201/eid2505.181247 PMC6478197 30807268

[68] J. Mariën , Data from: Density-dependence and persistence of Morogoro arenavirus transmission in a fluctuating population of its reservoir host. Dryad. 10.5061/dryad.0g22962. Deposited 19 December 2019. 31545505

[69] G. C. Milne , Climate-driven host ecology as a framework for zoonotic disease risk: Understanding transferability across arenavirus systems. Github. https://github.com/gcmilne/lassa-popdyn. Deposited 10 September 2026. 10.1073/pnas.2625913123 PMC13624649 42789295

