# Supplementary material for: Climate-driven host ecology as a framework for zoonotic disease risk: Understanding transferability across arenavirus systems
Source: Proc Natl Acad Sci U S A. 2026 Sep 25;123(39):e2625913123. doi: 10.1073/pnas.2625913123 (PMC13624649; doi:10.1073/pnas.2625913123)
Supplement: Supplementary file 1 [file pnas.2625913123.sapp.pdf]

PNAS

Supporting Information for

Climate-driven host ecology as a framework for zoonotic disease risk: understanding transferability across arenavirus systems

Gregory C. Milne, Lauren A. Attfield, Joachim Mariën, Lucinda Kirkpatrick, Herwig Leirs, Kate E. Jones, Christl A. Donnelly, and David W. Redding

Gregory C. Milne, David W. Redding.
E-mail: gregory.milne@nhm.ac.uk, david.redding@nhm.ac.uk

This PDF file includes:

Supporting text
Figs. S1 to S17
Tables S1 to S4
SI References

Supporting Information Text

1. Supplementary methods

A. Climate data. Daily precipitation and temperature data (1, 2) were extracted at the trapping site (3). To account for erroneous temperature data, we compared each daily value to a long-term mean (1991–2020) (4). Missing daily values and daily values with ≥60% absolute difference from the long-term mean were interpolated using neighbouring daily values. 28-day rolling averages were calculated for precipitation and temperature separately, matching the temporal resolution of the trapping data (3). These were subsequently decomposed into trend, seasonal, and residual (hereafter 'variable') components using seasonal-trend decomposition via locally estimated scatterplot smoothing (LOESS) (STL) (5), with a five-year trend window and a periodic seasonal window.

B. Statistical models. Bayesian hierarchical generalized linear models (GLMs) were formulated using long-term capture mark recapture data (3) to describe the demographic processes of body weight change, pregnancy, and survival. The vector of covariates, $\boldsymbol{X}$, for each model was: weight (w), population size ($N(t)$), temperature trend (t_{trend}), temperature seasonality (t_{seas}), temperature variability (t_{var}), precipitation trend (p_{trend}), precipitation seasonality (p_{seas}), and precipitation variability (p_{var}),

$$\boldsymbol{X}(t) = \{w, N(t), t_{\text{trend}}(t), t_{\text{seas}}(t), t_{\text{var}}(t), p_{\text{trend}}(t), p_{\text{seas}}(t), p_{\text{var}}(t)\} \qquad [1]$$

with corresponding vector of coefficients

$$\boldsymbol{b} = \{b_w, b_N, b_{t_{\text{trend}}}, b_{t_{\text{seas}}}, b_{t_{\text{var}}}, b_{p_{\text{trend}}}, b_{p_{\text{seas}}}, b_{p_{\text{var}}}\}. \qquad [2]$$

Labelling the elements of the vectors of covariates and coefficients as $\boldsymbol{X} = \{X_1, X_2, ..., X_8\}$ and $\boldsymbol{b} = \{b_1, b_2, ..., b_8\}$, the demographic process models took the form

$$g(\mu) = a + b_1 X_1 + b_2 X_2(t) + \sum_{i=3}^{8} b_i \sum_{k=1}^{K} \omega_k X_i(t - L_{i,k}) \qquad [3]$$

where $g(\mu)$ is the link function (logit(p) for the Bernoulli processes of survival and pregnancy, and μ for the mean of the continuous process of body weight change), $L_{i,k}$ is the kth temperature/precipitation lag corresponding to covariate i, and ω_k is that corresponding lag combination's assigned weight (see Section B.1).

The effect of body size on individual-level recruitment could only be observed in female rodents (via observable pregnancy and lactation). Therefore, body growth, pregnancy, and survival were only modelled directly in female rodents, with a separate model fit to estimate corresponding male population size. Coefficients for each demographic process are indicated with subscripts: B for body weight change, S for survival, P for pregnancy, and M for male population size (see Section B.5).

B.1. Climate lags. We introduced lags to the decomposed climate data ($X_3, ..., X_8$ in Eq. 1) (1, 2). We defined temperature lags $l_T = \{0, 28, ..., 168\}$ and precipitation lags $l_P = \{0, 28, ..., 168\}$ (units in days), giving $K = |l_T||l_P| = 7 \times 7$ lag combinations for each demographic process model. Lag weights, ω, associated with each lag combination were defined as a $(K-1)$ simplex, where $\omega_k \geq 0$ for $k \in \{1, ..., K\}$ and $\sum_{k=1}^{K} \omega_k = 1$. A uniform Dirichlet prior distribution (with all concentration parameters equal to one) was placed on this simplex ($\boldsymbol{\omega} \sim \text{Dir}(\boldsymbol{\alpha} = \{1, 1, ..., 1\})$). Lag weights $\boldsymbol{\omega}$ were estimated separately for each model, contributing to model likelihood through a weighted sum (Eq. 3). In Eq. 3, $L_{i,k} =$ takes the value of one of the $\{l_T, l_P\}$ pair corresponding to the kth combination of temperature-precipitation lags. If i corresponds to a temperature covariate ($i \in \{3, 4, 5\}$), then $L_{i,k}$ takes the value of the temperature lag, while if i corresponds to a precipitation covariate ($i \in \{6, 7, 8\}$) then $L_{i,k}$ is the paired precipitation lag.

B.2. Body weight change. Changes in body weight were observed by identifying consecutive individual capture records and recording the weight at all captures. Subsequent trapping sessions were defined as 3–5 weeks after the initial capture to allow for variability from the 28-day mean time between trapping sessions (3). An individual's body weight Y at time $t+1$ given it had a body weight w at time t was modelled as a heteroskedastic normal distribution

$$Y \sim \text{N}(\mu_B(w, t), \sigma_B(w)), \quad \text{where} \quad \sigma_B(w) = \exp(a_\sigma + b_\sigma w), \qquad [4]$$

with the mean of the distribution μ_B described as in Eq. 3 ($g(\mu_B) = \mu_B$), noting that the individual's body weight at time t is the first covariate in the vector of covariates ($w = X_1$). The probability density function of Y is then given by

$$p_B(y|w, t) = \frac{1}{\sigma_B(w)\sqrt{2\pi}} \exp\left(-\frac{1}{2}\left(\frac{v - \mu_B(w, t)}{\sigma_B(w)}\right)^2\right). \qquad [5]$$

B.3. Survival. Survival, whether an individual of body weight w at time t survives to time $t+1$, was modelled as a random variable drawn from a Bernoulli distribution with probability of success (survival) p_S. Since survival could not be directly observed from the data, we first modelled the probability of recapture, p_R, using the model outlined in Eq. 3 with a logit-link function for $g(\mu)$. We then related p_S to p_R by conditioning on whether the individual is recaptured or not:

$$\begin{aligned} p_S(w,t) &= \mathbb{P}(\text{alive at } t+1) \\ &= p_R(w,t) + \mathbb{P}(\text{alive at } t+1|\text{ never recaptured after } t)\cdot(1-p_R(w,t)). \end{aligned} \quad [6]$$

Since $\mathbb{P}(\text{alive at } t+1|\text{ never recaptured after } t)$ could not be known from the data, we defined this as $\kappa(w,t)$: the probability that an individual captured at time t of weight w is still alive at time $t+1$ despite never being recaptured (allowing for factors such as migration and stochasticity). It then follows that

$$p_S(w,t) = \kappa(w,t) + (1-\kappa(w,t))p_R(w,t). \quad [7]$$

Since it would not be possible to distinguish between κ and p_R if κ were allowed to vary by weight and time, κ was assumed constant (i.e., $\kappa(w,t)=\kappa$) and calibrated to obtain stable population dynamics (see Section F.4).

B.4. Pregnancy. We modelled pregnancy as the probability of a (not pregnant) female of weight w at time t being pregnant at time $t+1$. While recent pregnancy was observable from the data (3), inclusion of these individuals would bias estimates of the effect of weight on the probability of pregnancy. To avoid this, the data were restricted to females who were not pregnant at time t but were pregnant or lactating when captured in the next trapping session at time $t+1$ (3–5 weeks after initial capture), or lactating but not pregnant when captured in the subsequent trapping session at time $t+2$ (6–10 weeks after initial capture). This strategy was informed by the known reproductive timings of *M. natalensis*, of which the average gestation period is approximately 21 days, with visible pregnancy apparent in the second half of this period, followed by lactation for approximately 21 days (6). Similarly to survival, pregnancy was modelled using a Bernoulli likelihood and a logit-link function (Eq. 3).

B.5. Male population size. The integral projection model (IPM) models the female portion of the population (see Section F). However, since the demographic process models were fit using total population size (males and females) as a covariate (X_2 in Eq. 1), it was necessary to estimate total population size to ensure correct calibration of density dependence in the IPM.

To achieve this, we formulated and compared Bayesian GLMs to estimate male population size, N_m, as a function of the observed, time-specific female population size, $N_f(t)$, using a zero-truncated normal distribution (see Section D for model details). The best-fitting model (see Section 2) was of the form:

$$N_m \sim \text{TN}(\mu_M, \sigma_M^2, 0, \infty), \quad \text{where} \quad \mu_M = a_M + b_M N_f(t) + u_M. \quad [8]$$

Here, a_M is the global intercept, b_M is the effect of female population size, and u_M represents the random intercept for month (included to capture the time-dependent relationship between male and female population sizes). The standard deviation σ_M captures residual variation in the male population size. The distribution is truncated at zero to ensure no non-negative values for number of males N_m. By estimating this relationship, the IPM could dynamically update total population size at each time step, maintaining accurate density dependence (see Section F).

C. Fitting statistical models. The four statistical models of body weight change, survival, pregnancy, and male population size were fit in Stan (7) using a No-U-Turn Sampler (NUTS) (8) Hamiltonian Monte Carlo algorithm, with prior distributions as specified in Table S1. The observations x_j of a given covariate were transformed to unit scale (mean of 0, standard deviation of 1) by $\hat{x}_j = \frac{x_j - \bar{x}_j}{\sigma_{x_j}}$, where $\bar{x}_j$ represents the mean across measured values of x_j and σ_{x_j} the standard deviation across measured values of x_j. For models of survival and pregnancy we estimated the coefficients a and $\boldsymbol{b}$, and lag weights $\boldsymbol{\omega}$ (Eq. 3), while for body weight change we estimated these parameters plus a_σ and b_σ (Eq. 4; Table S1). Since $\boldsymbol{\omega}$ is comprised of K elements (denoting the total number of lag combinations; Section B.1), model estimation provided K posterior distributions for $\boldsymbol{\omega}$ for each demographic process. Hence, we estimated $K+9$ parameters for survival and pregnancy and $K+11$ for body weight change. For male population size, we estimated four parameters (a_M, b_M, u_M, and σ_M^2; Eq. 8). Models were fit to their respective datasets using four independent Markov chain Monte Carlo (MCMC) chains run for 2,000 iterations each, discarding the first 50% of iterations as burn-in. Convergence was assessed by ensuring $\hat{R}=1$ (7) and effective sample size $\hat{N} \geq 1000$ (9), and by visually inspecting MCMC trace plots. Correlation between posterior parameter values was assessed via cross-correlation plots.

D. Model comparison. For each demographic process (body weight change, survival, pregnancy), we fit models containing every possible combination of the six climate predictors ($X_3, .., X_8$ in Eq. 3), giving $2^6 = 64$ models for each demographic process. For each model M_i ($i \in \{1, 2, ...64\}$) we then derived via bridge sampling (10) an approximation of the marginal log-likelihood,

$$p(y|M_i) = \int p(y|\theta, M_i)p(\theta|M_i)d\theta \quad [9]$$

where $p(\boldsymbol{y}|\theta, M_i)$ is the likelihood of the parameters θ in the model M_i (given observations $\boldsymbol{y}$), and $p(\theta|M_m)$ is the prior distribution. The relative evidence for one model over another was then computed as a Bayes factor,

$$F(M_i, M_j) = \frac{p(\boldsymbol{y}|M_i)}{p(\boldsymbol{y}|M_j)}. \quad [10]$$

For male population size (see Section B.5), we estimated four models of varying complexity using the R package *brms* (9). All models contained female population size as a coefficient. Model 1 contained a fixed intercept. Model 2 contained a random intercept for month of sampling. Model 3 had a fixed intercept and a random coefficient for month. Model 4 had a random intercept plus a random coefficient for month. After model fitting, we made pairwise comparisons of each model's predictive accuracy using approximate leave-one-out cross-validation (LOO-CV) on expected log-predictive densities (ELPD)(9), with higher ELPD indicating better performance. If there was no difference in the ELPDs of two competing models, the simpler model was preferred in line with the principle of parsimony.

E. Posterior predictive checks. Posterior predictive checks (PPCs) were performed to assess the ability of each demographic model to capture relevant aspects of the data (e.g., mean, standard deviation). For each model, data for PPCs were generated by simulating from the posterior predictive distribution. The posterior predictive distribution for replications $\boldsymbol{y}_{\text{rep}}$ of the observed data $\boldsymbol{y}$ given model parameters θ can be expressed as

$$p(\boldsymbol{y}_{\text{rep}}|y) = \int p(\boldsymbol{y}_{\text{rep}}|\theta)p(\theta|\boldsymbol{y})d\theta. \quad [11]$$

Note that the notation $\boldsymbol{y}_{\text{rep}}$ indicates that the same predictors $\boldsymbol{X}$ are used to simulate from the posterior predictive distribution as are used to fit the model.

F. Integral projection model.

F.1. Model structure. We constructed an age- and weight-structured IPM to simulate population and infection dynamics of *M. natalensis* over discrete time steps $t = \{0, 1, ..., t_{\max}\}$ (with each time step representing 28 days to match the data's temporal resolution (3)), based on the fitted demographic process models of body weight change, survival, and pregnancy (Figure S1, Section B). Estimated parameters are given in Table S1, while model functions and fixed parameters are given in Table S2.

We denote the density of individuals of weight w and age a in the population at time t as $D(w, t, a)$, where $a \in 1, 2$ denotes the sub-adult class and $a \geq 3$ denotes the adult class. The density of pregnant individuals is denoted $J(w, t)$ (so note that $J(w, t) \leq \sum_{a \geq 3} D(w, t, a)$, since all pregnant individuals are adults). Individuals at time t determined to become pregnant (with probability $p_P(w, t)$; Section B.4) move to compartment J at time $t + 1$. It was then assumed that if the pregnant individual survives to $t + 2$, they recruit l female offspring where l is a constant (set to 5 (3)). Offspring follow a body weight distribution given by $v(w)$ which was parameterized with data (11). Consistent with evidence showing that female *M. natalensis* can become pregnant approximately 4 weeks after giving birth (12), the model allows individuals in compartment J at time t to leave the compartment at time $t+2$. Individuals are born into the first sub-adult class ($a = 1$) and reach the adult class ($a = 3$) at three time steps (i.e, 84 days), meaning they can then become pregnant and can give birth to offspring at the fifth time step (140 days), approximately matching average age of first litter estimates from empirical data (130 days (12)). For ease, we will define $D(w, t)$ as the marginalised density of body weight w at time t across both age classes (i.e., $D(w, t) = \sum_a D(w, t, a)$). Noting the pregnancy probability p_P is only applied to the adult population ($a \geq 3$) and that sub-adults ($a = 1, 2$) are governed by the same growth and survival functions as adults (conditioned on weight), the model is then given by the integral projection equations:

$$\begin{aligned}
J(w, t+1) &= \int_0^\infty p_B(w|y, t)p_S(y, t)p_P(y, t) \left(\sum_{a \geq 3} D(y, t, a) - J(y, t) \right) dy \\
D(w, t+1, 0) &= \int_0^\infty l p_S J(y, t) v(w) \, dy \\
D(w, t+1, a+1) &= \int_0^\infty p_B(w|y, t)p_S(y, t)D(y, t, a) \, dy \quad \text{for } a \geq 1
\end{aligned} \quad [12]$$

To implement the model computationally, Eq. 12 was discretized by converting probability density functions, $p_G(w, t)$ and $v(w)$, into probability mass functions and converting integrals into sums over a set of possible weights $W = \{1, 2, 3, ..., 100\}$ (in grams; chosen to capture the full possible range of weights since observed rodents did not exceed 96 grams (3)), giving the discretized form of the model:

$$J(w,t+1) = \sum_{y\in W} p_B(w|y,t)p_S(y,t)p_P(y,t)\left(\sum_{a\geq 3} D(y,t,a) - J(y,t)\right)$$
$$D(w,t+1,0) = \sum_{y\in W} lp_S(y,t)J(y,t)v(w) \quad [13]$$
$$D(w,t+1,a+1) = \sum_{y\in W} p_B(w|y,t)p_S(y,t)D(y,t,a) \quad \text{for } a\geq 1$$

where now the compartments $D(w,t,a)$ and $J(w,t)$ refer to the number, rather than density, of individuals of weight w at time t. Note that all adult age classes ($a \geq 3$) are treated equally and therefore the system can be implemented with just three age classes ($a = 1$, $a = 2$, and $a \geq 3$) rather than tracking ages of adults.

F.2. Infection dynamics. The IPM was expanded to include pathogen transmission dynamics in a compartmental model of the form: susceptible (S), horizontally infected (H), vertically infected (V), and recovered (R) (Figure S1). This structure was motivated by empirical data and previous models of arenavirus dynamics wherein vertically infected individuals remain chronically infectious for life whereas horizontally infected individuals are transiently infectious and recover in approximately 30 days (13). The model takes the form,

$$\frac{dS}{dt} = b(S + (1-\varphi)(H+V)) + \eta R - \beta(t)S(H+V) - mS$$
$$\frac{dH}{dt} = \beta(t)S(H+V) - \gamma H - mH$$
$$\frac{dV}{dt} = \varphi b(H+V) - mV \quad [14]$$
$$\frac{dR}{dt} = bR + \gamma H - \eta R - mR$$

where $\beta(t)$ is the horizontal transmission rate (estimated; Section G), γ is the recovery rate following horizontal transmission (28^{-1} days (13)), m is the intrinsic mortality rate, b is the intrinsic birth rate, φ is the probability of vertical transmission between an infected individual and its offspring (estimated; Section G), and η is the rate of loss of transient maternal antibodies acquired from an offspring birthed by a recovered mother (28^{-1} days (14)) (Tables S1, S2).

To nest this compartmental model within the IPM, it was first necessary to estimate the number of horizontal infections between time steps. We estimated the horizontal force of infection, $\lambda(t)$, using a fourth-order Runge-Kutta (RK4) method as follows. At time step t, the susceptible (S_t), horizontally-infected (H_t), and vertically-infected (V_t) populations are defined by integrating over their respective weight distributions (e.g., $H_t = \int_0^\infty D_H(w,t)dw$). By assuming no demographic or infection changes between time steps, the number of new horizontal infections between time t and $t+\tau$ (where $\tau \in (0,1]$), $K_t(\tau)$, can be shown to follow a susceptible-infected (SI) version of Eq. 14:

$$\frac{dS}{d\tau} = -\beta SI,$$
$$\frac{dI}{d\tau} = \beta SI, \quad [15]$$
$$\frac{dK_n}{d\tau} = \beta SI,$$

where $S(\tau = 0) = S_t$, $I(\tau = 0) = H_t + V_t$, and $K_t(\tau = 0) = 0$. Using RK4, we estimate $K_t(\tau = 1)$ with the following slopes:

$$k_1 = \beta(t)S_tI_t,$$
$$k_2 = \beta(t)\left(S_t - \frac{k_1}{2}\right)\left(I_t + \frac{k_1}{2}\right),$$
$$k_3 = \beta(t)\left(S_t - \frac{k_2}{2}\right)\left(I_t + \frac{k_2}{2}\right), \quad [16]$$
$$k_4 = \beta(t)(S_t - k_3)(I_t + k_3).$$

The total number of new infections over the time step is

$$E_t = K_t(1) = \frac{1}{6}(k_1 + 2k_2 + 2k_3 + k_4). \quad [17]$$

The force of infection on each susceptible individual (assumed to be independent of body weight) is then given by

$$\lambda(t) = \frac{E_t}{S_t} \tag{18}$$

The compartmental model is then nested within the (discretized) IPM:

$$\begin{aligned}
B_S(t+1) &= l \sum_{y \in W} p_S(y,t) J_S(y,t) \\
B_I(t+1) &= l \sum_{y \in W} p_S(y,t) J_{H+V}(y,t) \\
B_R(t+1) &= l \sum_{y \in W} p_S(y,t) J_R(y,t)
\end{aligned} \tag{19}$$

$$\begin{aligned}
D_S(w,t+1,1) &= \big(B_S(t+1) + (1-\varphi) B_I(t+1)\big) v(w) \\
D_H(w,t+1,1) &= 0 \\
D_V(w,t+1,1) &= \varphi B_I(t+1) v(w) \\
D_R(w,t+1,1) &= B_R(t+1) v(w)
\end{aligned} \tag{20}$$

$$\left.\begin{aligned}
D_S(w,t+1,a+1) &= \textstyle\sum_{y \in W} p_B(w|y,t) p_S(y,t) \big((1-\lambda(t)) D_S(y,t,a) + \chi_{\{1\}}(a) D_R(y,t,a)\big) \\
D_H(w,t+1,a+1) &= \textstyle\sum_{y \in W} p_B(w|y,t) p_S(y,t) \lambda(t) \sum_{a \geq 3} D_S(y,t,a) \\
D_V(w,t+1,a+1) &= \textstyle\sum_{y \in W} p_B(w|y,t) p_S(y,t) D_V(y,t,a) \\
D_R(w,t+1,a+1) &= \textstyle\sum_{y \in W} p_B(w|y,t) p_S(y,t) \big((1-\chi_{\{1\}}(a)) D_R(y,t,a) + D_H(y,t,a)\big)
\end{aligned}\right\} \text{ for } a \geq 1 \tag{21}$$

$$\begin{aligned}
J_S(w,t+1) &= \sum_{y \in W} p_P(y,t) \big(1-\lambda(t)\big) p_B(w|y,t) p_S(y,t) \sum_{a \geq 3} D_S(y,t,a) \\
J_{H+V}(w,t+1) &= \sum_{y \in W} p_P(y,t) p_B(w|y,t) p_S(y,t) \sum_{a \geq 3} \big(\lambda(t) D_S(y,t,a) + D_V(y,t,a)\big) \\
J_R(w,t+1) &= \sum_{y \in W} p_P(y,t) \Big(p_B(w|y,t) p_S(y,t) \sum_{a \geq 3} D_R(y,t,a) + p_B(w|y,t) p_S(y,t) \sum_{a \geq 3} D_H(y,t,a)\Big).
\end{aligned} \tag{22}$$

In this system of equations, B_S, B_I, and B_R are the number of susceptible, infected, and recovered (maternally-antibody positive) juveniles, respectively, entering the population, and subscripts for D and J denote infection status within the whole population and the pregnant sub-population, respectively. $\chi_{\{1\}}(a)$ denotes the indicator function which takes the value of 1 if $a = 1$ (which here causes movement of sub-adults with maternal antibodies moving from recovered to susceptible infection status) and 0 otherwise. Modelled (female) population size can then be given by

$$N_f(t) = \sum_{w \in W} \sum_a \big(D_S(w,t,a) + D_H(w,t,a) + D_V(w,t,a) + D_R(w,t,a)\big) \tag{23}$$

F.3. Calibrating density dependence. To ensure proper calibration of density dependence while simulating the IPM using Eqs. (19) to (22), $N_f(t)$ (Eq. 23) had to be adjusted to total population size. This ensured no discrepancy between the total population size data used to fit demographic process models (X_2 in Eqs. 1 and 3) and the population covariates used as inputs to fitted demographic process models during IPM simulation. At every model t, $N_f(t)$ was used to derive an estimated total population size, $N_{m,f}(t)$, by

$$N_{m,f}(t) = N_f(t) + N_m(t). \tag{24}$$

where N_m was predicted at each time step of the IPM using Eq. 8 (using fitted parameter values for a_M, b_M, and σ_M). Before input into fitted demographic process models (Eq. 3), $N_{m,f}(t)$ was further adjusted to bring the absolute modelled population size in line with the mean observed population size,

$$N(t) = \left(\frac{\bar{N}}{2N_0}\right) N_{m,f}(t) \tag{25}$$

where $\bar{N}$ is the mean observed Minimum Number Alive (male and female) (3) and N_0 is the initial modelled female population size (at $t = 0$). The term $2N_0$ accounts for the (assumed 1:1) sex ratio which was necessary to include because the demographic process models were fit to data on total population size (Section B) whereas the IPM only explicitly considered females but requires total population size as an input (Eqs. 8, 24, 25).

F.4. Calibrating survival. We calibrated κ to obtain the probability of survival, p_S, from the probability of recapture, p_R (Section B.3, Eqs. 6, 7), as follows. The IPM was simulated for $N = 1000$ random posterior draws of the fitted statistical model parameters (Section C) to obtain predictions of the time-specific modelled population size, $N(t)$ (Eq. 23). For each joint posterior draw, the IPM was simulated and values of κ interpolated to obtain a modelled population size that was within 1% of the initial population size at $t = 0$, N_0. In subsequent model fitting (Section G) and posterior prediction (Section H), κ was set to the median of this estimated distribution which ensured stable population dynamics.

G. Fitting transmission parameters.

A Bayesian adaptive grid search algorithm was developed to approximate the posterior distributions of transmission parameters φ and β, by fitting to Morogoro arenavirus seroprevalence data collected 2010–2016 (withholding 2017 data for validation) (14). We set prior distributions $\varphi \sim Beta(20, 2)$ (15, 16) and $\beta \sim$ U$(0.00001, 0.0015)$. The parameter space was discretized into a 20×20 coarse grid $\mathbf{C}$ over the intervals $\varphi \in [0.01, 0.99]$, $\beta \in [0.0001, 0.0015]$. For $i = \{1, 2, ..., N\}$ (where $N = 1000$) random independent draws from the demographic model posterior distributions, this coarse grid was adjusted by some random uniform noise ε_i in both dimensions to obtain new grid values $\mathbf{C}^{(i)} = \mathbf{C} + \varepsilon_i$, where ε_i for each parameter was uniform on $(-\frac{d}{2}, +\frac{d}{2})$, where d is the (equal) difference between consecutive parameter values in the grid $\mathbf{C}$. The IPM was simulated using the sampled demographic parameter values for each of the coarse grid infection parameter values to derive modelled seroprevalences for sub-adults and adults by $\big(N(t) - S(t)\big)/N(t)$. These modelled seroprevalence outputs were compared to empirical data using joint binomial log-likelihood distributions, which were combined with log-prior distributions to obtain unnormalised log-posterior densities. Log-sum-exp transformation was used to transform unnormalised log-posterior densities into importance sampling weights. Parameter combinations with importance weights below a threshold $\tau < 1 \times 10^{-7}$ were discarded and the remainder used to construct a finer grid spanning this high density region of parameter space. For each demographic draw, this fine grid was re-evaluated to obtain a smoother posterior probability surface which was used to generate posterior predictions (see Section H).

H. Posterior predictions.

To propagate uncertainty from previously estimated model parameters into downstream inference, we adopted a Bayesian melding framework (17, 18). Specifically, we first obtained posterior samples from four upstream statistical models (denoted collectively as Φ; Section C) by fitting to demographic and climate data, $\mathcal{D}_1$. We then sought to estimate two additional transmission dynamics parameters, denoted collectively as ϕ, by combining information from these upstream models with a second data source, $\mathcal{D}_2$, comprising seroprevalence observations (Section G).

For each upstream posterior sample $\Phi^{(i)} \sim p(\Phi|\mathcal{D}_1)$, we evaluated the conditional posterior distribution over ϕ given the seroprevalence data:

$$p(\phi|\Phi^{(i)}, \mathcal{D}_2) \propto p(\phi) \times \mathcal{L}_2(\mathcal{D}_2|\Phi^{(i)}, \phi), \tag{26}$$

where $\mathcal{L}_2$ is the likelihood of the seroprevalence data under model parameters $(\Phi^{(i)}, \phi)$, and $p(\phi)$ is the prior distribution over the transmission parameters. This conditional posterior distribution was approximated over a 2D grid for each $\Phi^{(i)}$ (see Section G) to obtain a posterior density function $PD_i(\phi) = p(\phi|\Phi^{(i)}, \mathcal{D}_2)$. The joint posterior distribution over both upstream and transmission parameters can then be approximated by:

$$p(\Phi^{(i)}, \phi^{(j)}) \approx p(\Phi^{(i)}|\mathcal{D}_1) \cdot p(\phi^{(j)}|\Phi^{(i)}, \mathcal{D}_2). \tag{27}$$

To sample efficiently from this joint distribution, we used importance resampling at two levels. First, we re-weighted the upstream samples $\Phi^{(i)}$ based on the concordance between modelled and empirical seroprevalence. Specifically, we marginalised over each conditional posterior $PD_i(\phi)$ to compute an integrated weight:

$$w_i \propto \int p(\phi|\Phi^{(i)}, \mathcal{D}_2)\, d\phi \approx \sum_j PD_i(\phi^{(j)}), \tag{28}$$

where $PD_i(\phi^{(j)})$ are unnormalised posterior densities. To ensure numerical stability, we computed this sum on the log scale using log-sum-exp transformation:

$$\log w_i = \log \sum_j \exp\left(\log PD_i(\phi^{(j)}) - \max_j \log PD_i(\phi^{(j)})\right) + \max_j \log PD_i(\phi^{(j)}), \tag{29}$$

These weights w_i were then exponentiated and normalised to sum to 1. The resulting normalised weights reflect the compatibility of each upstream draw $\Phi^{(i)}$ with the transmission dynamics implied by the seroprevalence data $\mathcal{D}_2$. Second, for each selected draw $\Phi^{(i)}$, we generated samples of the transmission parameters ϕ by resampling from the corresponding conditional posterior surface $PD_i(\phi)$. Specifically, we treated the unnormalised log posterior densities $\log PD_i(\phi^{(j)})$ over the grid as importance weights, stabilised them using a log-sum-exp transformation, and normalised them to define a discrete probability distribution over ϕ. This ensures that transmission parameter values are sampled with probability proportional to their posterior support, conditional on the selected upstream model $\Phi^{(i)}$. The Bayesian melding procedure thus proceeds as follows:

- For each $\Phi^{(i)}$, compute the conditional posterior surface $PD_i(\phi)$ over a grid.
- Marginalise over each $PD_i(\phi)$ to obtain an importance weight w_i for $\Phi^{(i)}$ (Eq. 29).

- Draw $N = 1000$ samples of (Φ, ϕ) by:
  1. Sampling $\Phi^{(i)}$ with probability proportional to w_i.
  2. Sampling $\phi^{(j)}$ from the discretized grid conditional on $\Phi^{(i)}$, with probability proportional to $PD_i(\phi^{(j)})$, i.e., the normalised posterior density for ϕ conditional on the selected $\Phi^{(i)}$.

Posterior predictive simulations were summarized by medians and 95% credible intervals (0.5, 0.025, and 0.975 quantiles).

I. Model recalibration for Nigeria.

I.1. Posterior predictions. We re-calibrated the fitted model to Nigeria to determine the extent to which the seasonal patterns of Lassa fever outbreaks could be captured. Specifically, we focussed on the five Nigerian states with the highest burden of Lassa fever (Bauchi, Ebonyi, Edo, Ondo, and Taraba) (19) and extracted data for 2018–2025 due to improvements in testing capacity in early 2018 (20). We extracted daily temperature and precipitation data (1, 2) at the centroid of each state, decomposed these as previously (Section B), and for each state recalibrated survival adjustments (κ) using the state-specific climate data (Section F.4). We then made posterior predictions of infection dynamics for each state by simulating the model with the $N = 1000$ (Φ, ϕ) draws (Section H), substituting the Morogoro climate data for the Nigerian state-specific climate data. For each Nigerian state s, we predicted the time-specific zoonotic hazard H as the number of infected rodents:

$$H_s(t) = \sum_{w} \Big(D_{H,s}(w,t) + D_{V,s}(w,t) \Big) \qquad [30]$$

which we transformed into a relative metric by

$$H_s^*(t) = \frac{H_s(t)}{\max_t(H_s(t))} \qquad [31]$$

I.2. Lassa fever case data. We compared predictions to weekly reports of confirmed Lassa fever cases from the Nigeria Centre for Disease Control and Prevention (19). Let $Y_s(d)$ be the reported cases in state s on day d and let us assume, due to imperfect sampling, that the reported case numbers are not the true cases numbers. We can then estimate uncertainty in the true case numbers by:

$$\hat{Y}_s(d) \sim \text{Poisson}(\lambda = Y_s(d)) \qquad [32]$$

To make model estimates comparable to the data, case data were transformed to obtain an infection time series by sampling from an inferred Lassa fever incubation period distribution p_I:

$$I_s(d) = \sum_{i=1}^{30} \hat{Y}_s(d+i) p_I(i) \qquad [33]$$

To make observed case data comparable to model predictions, this was transformed into a relative metric by:

$$I_s^*(d) = \frac{I_s(d)}{\max_d(I_s(d))} \qquad [34]$$

p_I was inferred by collating published data on nosocomial infections of Lassa fever. Although infections in a healthcare setting are caused by human-to-human transmission rather than zoonotic transmission—and thus one might expect differences in virulence or viral dose that could lead to different incubation periods—we emphasise that we used the only available data to make these estimates. Papers were considered if they appeared in a PubMed or Scopus search carried out in September 2025 for "("Lassa fever") AND ("nosocomial" OR "hospital infection")". Individual cases of Lassa fever were included if they:

- were confirmed through laboratory testing
- were believed to have been infected in a healthcare setting
- had a finite and informative period for exposure (time window of 0–28 days)

For each included case ($N = 30$) we extracted data on study location, earliest and latest possible date of exposure, earliest and latest possible date of symptom onset, and fatality (including date of fatality if recorded) (Table S3).

We used the Metropolis-Hastings (MCMC) algorithm to fit a log-normal distribution (Lognormal(μ, σ^2)) to the observed incubation period windows with an uninformative prior distribution. We took into account that the case data recorded possible windows of symptom onset rather than exact incubation periods by calculating likelihood as the probability of observing a set of incubation periods within the observed windows:

$$\ell(\mu, \sigma^2) = \prod_i \int_{w_i^- - \frac{1}{2}}^{w_i^+ + \frac{1}{2}} p(x; \mu, \sigma^2)\, dx = \prod_i \left(\text{erf}\Big(\frac{\log(w_i^+ + \frac{1}{2}) - \mu}{\sigma}\Big) - \text{erf}\Big(\frac{\log(w_i^- - \frac{1}{2}) - \mu}{\sigma}\Big) \right) \qquad [35]$$

where p denotes the probability density function of the log-normal distribution, i indexes the collated nosocomial cases, and w_i^- and w_i^+ denote the minimum and maximum (respectively) possible incubation period (in days) based on the hospital observations recorded. The addition and subtraction of $\frac{1}{2}$ is to account for the log-normal distribution being continuous while records are in discrete numbers of days.

2. Supplementary results

The best-performing model for growth (body weight change), as indicated by log marginal likelihoods, included coefficients for precipitation seasonality and precipitation trend. These coefficients were also present in the top 20.3% (13/64) of best-performing growth models (Figure S2). The best-performing and converging model for survival included precipitation seasonality and temperature trend. These coefficients were also present in the top 25% (16/64) of best-performing survival models (Figure S3). All 16 survival models including temperature seasonality did not converge (Figure S3), indicating the unsuitability of this variable for explaining survival. The best performing model for pregnancy included precipitation seasonality and precipitation variability. These coefficients were also present in the top 12.5% (8/64) of best-performing pregnancy models (Figure S4). Posterior distributions for the best-performing models are shown in Figure S5. Posterior estimates for climate lag weights were largely uninformative on the importance of specific climate lags (posterior distribution medians relatively uniform over prior distribution range of 0–168 days; Figure S6), indicating that demography integrates climate signals over extended periods rather than responding to specific past time points.

The model of male population size with the highest predictive accuracy, as indicated by expected log predictive densities, had a random intercept for month; this model outcompeted both simpler and more complex models (Table S4; Eq. 8).

Posterior predictive checks indicated good concordance between predicted and observed values for growth (Figure S7), survival (Figure S8), pregnancy (Figure S9), and male population size (Figure S10).

The posterior distributions of parameters for each demographic process model are shown in Figure S5. Posterior predictions of the contribution of each estimated parameter to the demographic process response is shown in Figure S11. Posterior predictions of the number of pregnancies are shown in Figure S13. The proportion of trapping sessions in which predicted seroprevalence overlaps observed seroprevalence is shown in Figure S12. Posterior predictions of the proportion of the modelled population in each epidemiological compartment are shown in Figure S14. Posterior predictions of prevalence compared to seroprevalence are shown in Figure S15; these results indicate that seroprevalence is a lagged indicator for prevalence (peaking $\sim$6.5 months after prevalence).

When assessing the ability of the median predicted seroprevalence (see main text) to capture 2017 hold-out data using a Bayesian GLM, we found moderately better predictive ability compared to an intercept-only null model (expected log predictive density difference 13.4 (expected log predictive density difference standard error 11.3)).

The median Lassa fever incubation period was estimated as $\log \mu = 7.50$ (95% CrI $(6.62, 8.55)$) days, with a logarithm of scale parameter $\sigma = 0.30$ $(0.22, 0.42)$. This gives a central 95% prediction interval for the incubation period of $(4.04, 14.08)$ days.

Figure S16 shows a comparison of the seasonal pattern of Lassa fever outbreaks to the seasonal patterns of both *M. natalensis* climatic/demographic drivers and the model-predicted number of infected sub-adult rodents. Figure S17 shows overlap in peak sizes for predicted and observed values for each of the five Nigerian states (panel A) and probabilities of outbreak size concordance at different thresholds (panel B).

Table S1. Estimated model parameter posteriors. Parameters and priors for demographic process models (body weight change, survival, recruitment) are unit-scaled. $\mathcal{N}(\mu, \sigma)$: normal prior with mean μ and standard deviation σ; $\mathcal{U}(a, b)$: uniform prior on (a, b); $\mathrm{Dir}(\alpha = 1)$: symmetric Dirichlet prior; Beta(a, b): beta prior with shape parameters a and b. CrI: credible interval.

Parameter	Description	Prior	Posterior (median, 95% CrI)
Body weight change			
$a_{B,\mu}$	Intercept, mean	$\mathcal{N}(0, 1)$	$0.214\ (0.185,\ 0.243)$
$b_{B,w}$	Coefficient, weight (mean)	$\mathcal{N}(0, 1)$	$0.782\ (0.763,\ 0.800)$
$b_{B,N}$	Coefficient, population size	$\mathcal{N}(0, 1)$	$-0.104\ (-0.125,\ -0.082)$
$b_{B,p_{\mathrm{trend}}}$	Coefficient, precip. trend	$\mathcal{N}(0, 1)$	$0.041\ (0.026,\ 0.056)$
$b_{B,p_{\mathrm{seas}}}$	Coefficient, precip. seasonality	$\mathcal{N}(0, 1)$	$0.775\ (0.704,\ 0.847)$
$a_{B,\sigma}$	Intercept, variance	$\mathcal{N}(0, 1)$	$-0.710\ (-0.733,\ -0.686)$
$b_{B,\sigma}$	Coefficient, variance	$\mathcal{N}(0, 1)$	$0.150\ (0.128,\ 0.173)$
ω_B	Climate lag weights	$\mathrm{Dir}(\alpha = 1)$	See Fig. S6
Survival			
a_S	Intercept	$\mathcal{N}(0, 2)$	$-0.404\ (-0.447,\ -0.358)$
$b_{S,w}$	Coefficient, weight	$\mathcal{N}(0, 1)$	$0.057\ (0.021,\ 0.094)$
$b_{S,N}$	Coefficient, population size	$\mathcal{N}(0, 1)$	$0.049\ (0.007,\ 0.089)$
$b_{S,t_{\mathrm{trend}}}$	Coefficient, temp. trend	$\mathcal{N}(0, 1)$	$-0.223\ (-0.258,\ -0.190)$
$b_{S,p_{\mathrm{seas}}}$	Coefficient, precip. seasonality	$\mathcal{N}(0, 1)$	$0.285\ (0.188,\ 0.383)$
ω_S	Climate lag weights	$\mathrm{Dir}(\alpha = 1)$	See Fig. S6
Recruitment			
a_P	Intercept	$\mathcal{N}(0, 3)$	$-5.00\ (-5.73,\ -4.37)$
$b_{P,w}$	Coefficient, weight	$\mathcal{N}(0, 2)$	$0.298\ (0.131,\ 0.472)$
$b_{P,N}$	Coefficient, population size	$\mathcal{N}(0, 2)$	$-1.74\ (-2.38,\ -1.18)$
$b_{P,p_{\mathrm{seas}}}$	Coefficient, precip. seasonality	$\mathcal{N}(0, 2)$	$3.58\ (2.89,\ 4.34)$
$b_{P,p_{\mathrm{var}}}$	Coefficient, precip. variability	$\mathcal{N}(0, 2)$	$0.839\ (0.342,\ 1.35)$
ω_P	Climate lag weights	$\mathrm{Dir}(\alpha = 1)$	See Fig. S6
Transmission			
β	Horizontal transmission rate	$\mathcal{U}(0.00001,\ 0.0015)$	$6.67 \times 10^{-4}\ (5.73 \times 10^{-4},\ 7.41 \times 10^{-4})$
φ	Vertical transmission probability	$\mathrm{Beta}(20, 2)$	$0.791\ (0.693,\ 0.889)$

Table S2. Fixed model parameters and functions. Parameters listed as "Estimated" are derived from model fitting; see referenced equations for details.

Parameter/function	Definition	Value	Ref.
Demographic functions			
$p_B(y\|w,t)$	Probability distribution of body weight change	Estimated	Eqs. 3, 5
$p_R(w,t)$	Probability of recapture	Estimated	Eq. 3
$p_S(w,t)$	Probability of survival	Estimated	Eqs. 3, 6, 7
$p_P(w,t)$	Probability of pregnancy	Estimated	Eq. 3
κ	Probability of being alive at $t+1$ despite non-recapture at t	Estimated	Eqs. 6, 7
Fixed parameters			
l	Female offspring per pregnancy	5	(3)
$v(w)$	Juvenile weight distribution	$\mathcal{N}(13, 0.3)$	(11)
γ	Recovery rate from horizontal infection	$(28\text{ days})^{-1}$	(13)
η	Seroreversion rate of maternal antibodies	$(28\text{ days})^{-1}$	(14)

Table S3. Data collated on nosocomial transmission of Lassa virus, used to estimate an incubation period distribution.

Location	Exposure period	Symptom onset period	Incubation period (days)	Fatal (date)	Ref
Jos, Plateau State, Nigeria	25/01/1970	03/02/1970	9	Yes (13/02/1970)	(21)
Jos, Plateau State, Nigeria	25/01/1970–13/02/1970	20/02/1970	7–26	No	(21)
Zorzor, Lofa County, Liberia	02/03/1972–18/03/1972	22/03/1972	4–20	No	(22)
Zorzor, Lofa County, Liberia	03/03/1972–19/03/1972	22/03/1972	3–19	No	(22)
Zorzor, Lofa County, Liberia	15/03/1972–19/03/1972	20/03/1972	1–5	Yes (04/04/1972)	(22)
Zorzor, Lofa County, Liberia	02/03/1972–19/03/1972	20/03/1972	1–18	No	(22)
Zorzor, Lofa County, Liberia	01/03/1972–13/03/1972	13/03/1972	3–12	NA	(22)
Zorzor, Lofa County, Liberia	01/03/1972–08/03/1972	17/03/1972	9–16	NA	(22)
Imo State, Nigeria	23/01/1989	29/01/1989	6	Yes (15/02/1989)	(23)
Imo State, Nigeria	25/02/1989	03/03/1989	6	Yes (15/03/1989)	(23)
Imo State, Nigeria	25/02/1989	03/03/1989	6	Yes (15/03/1989)	(23)
Imo State, Nigeria	25/02/1989	07/03/1989	10	No	(23)
Imo State, Nigeria	25/02/1989	07/03/1989	10	NA	(23)
Abakaliki, Ebonyi State, Nigeria	03/01/2012	08/01/2012	5	NA	(24)
Abakaliki, Ebonyi State, Nigeria	03/01/2012	08/01/2012–15/01/2012	5–12	NA	(24)
Abakaliki, Ebonyi State, Nigeria	03/01/2021	08/01/2012–15/01/2012	5–12	NA	(24)
Abakaliki, Ebonyi State, Nigeria	03/01/2021	08/01/2012–15/01/2012	5–12	NA	(24)
Abakaliki, Ebonyi State, Nigeria	03/01/2021	08/01/2012–15/01/2012	5–12	NA	(24)
Tanguiétta, Atakora Department, Benin	07/10/2014–14/10/2014	21/10/2014	7–14	Yes (NA)	(25)
Tanguiétta, Atakora Department, Benin	07/10/2014–14/10/2014	23/10/2014	9–16	Yes (NA)	(25)
Tchaourou, Borgou Department, Benin	03/01/2016	08/01/2016	5	No	(25)
Tchaourou, Borgou Department, Benin	03/01/2016	12/01/2016	9	No	(25)
Tchaourou, Borgou Department, Benin	03/01/2016	13/01/2016	10	Yes (NA)	(25)
Tchaourou, Borgou Department, Benin	03/01/2016	13/01/2016	10	No	(25)
Nigeria	28/12/2017	03/01/2018	6	Yes (14/01/2018)	(26)
Nigeria	28/12/2017	09/01/2018	12	Yes (14/01/2018)	(26)
Nigeria	28/12/2017	03/01/2018	6	Yes (04/01/2018)	(26)
Nigeria	28/12/2017	05/01/2018	8	No	(26)
Sierra Leone	04/11/2019	11/11/2019	7	Yes (NA)	(27)
Sierra Leone	04/11/2019	11/11/2019	7	No	(27)

Table S4. Predictive accuracy of four competing Bayesian models of male population size. Models are listed in order of performance. Metrics are in relation to the top performing model, with negative ELPD difference indicating poorer performance and SE differences quantifying uncertainty in ELPD. See Sections B.5 and D for a description of models. ELPD, expected log predictive density. SE, standard error.

	ELPD difference	SE difference
Model 2	0.0	0.0
Model 4	0.0	0.2
Model 1	-49.9	10.2
Model 3	-51.3	10.4

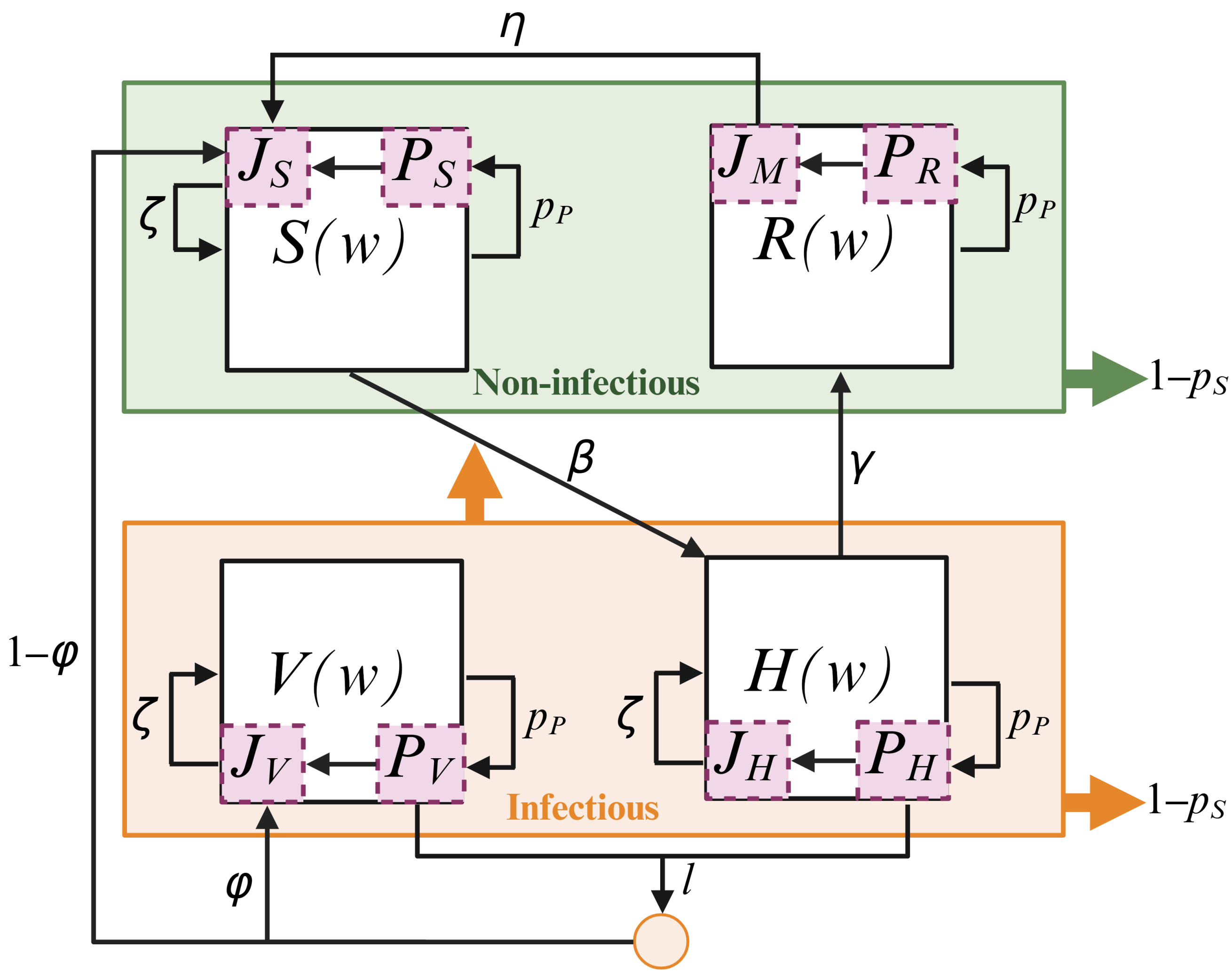

Fig. S1. Structure of the integral projection model. The population is divided into weight classes (denoted w), age classes (juveniles J and adults), and four epidemiological compartments representing susceptible (S), horizontally infected (H), vertically infected (V), and recovered (previously horizontally-infected, R) individuals. Adults become pregnant at probability p_P (transitioning to sub-compartment P), and all individuals die at probability $1 - p_S$. Each compartment is weight-structured, tracking a continuous distribution of body weights w; individuals transition between weight classes at each time step according to growth probability p_G (omitted from figure for clarity). Pregnant individuals produce l offspring (litter size) which enter the juvenile sub-compartment. Juveniles mature into adults at rate ζ; only adults are able to become pregnant. Pregnancies from infected compartments (H and V) have probability φ of producing vertically infected juveniles (J_V), with the remainder $(1 - \varphi)$ being susceptible (J_S). Recovered mothers (P_R) produce juveniles in a transiently maternal antibody-positive sub-compartment (J_M), transitioning at rate η to susceptible juveniles (J_S). Individuals in the infectious compartment group (H and V; orange box) contribute to infection of susceptible individuals via horizontal transmission rate β, with recovery from H to R occurring at rate γ.

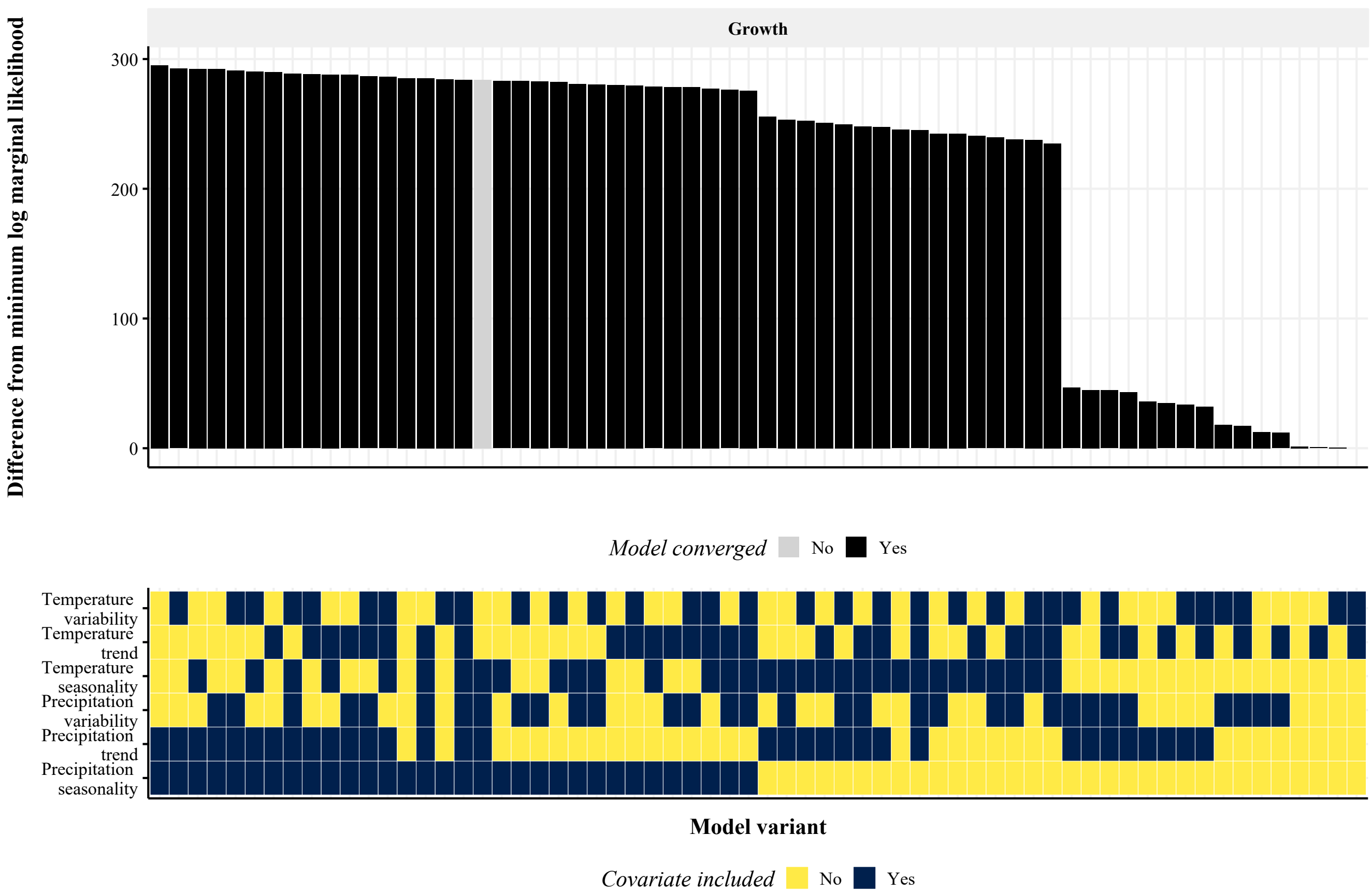

Fig. S2. Body weight change (growth) model performance for 64 model variants comprising all combinations of 6 climate predictors. The model estimates body weight at time $t + 1$ given demographic and climate covariates at time t. Models were fit to three decades of rodent trapping data (3) and climate data (1, 2) using a No-U-Turn Sampler (8) Hamiltonian Monte Carlo algorithm in Stan (7), with prior distributions as specified in Table S1. Approximate marginal log-likelihoods were calculated via bridge sampling (10). The bottom panel shows the covariates included in each model, while the top panel shows the corresponding performance of that model relative to the worst-performing model. Model variants are ordered from best (left) to worst (right) performing. Grey bars indicate models that did not converge ($\hat{R} \geq 1.05$ for ≥ 1 parameter(s)).

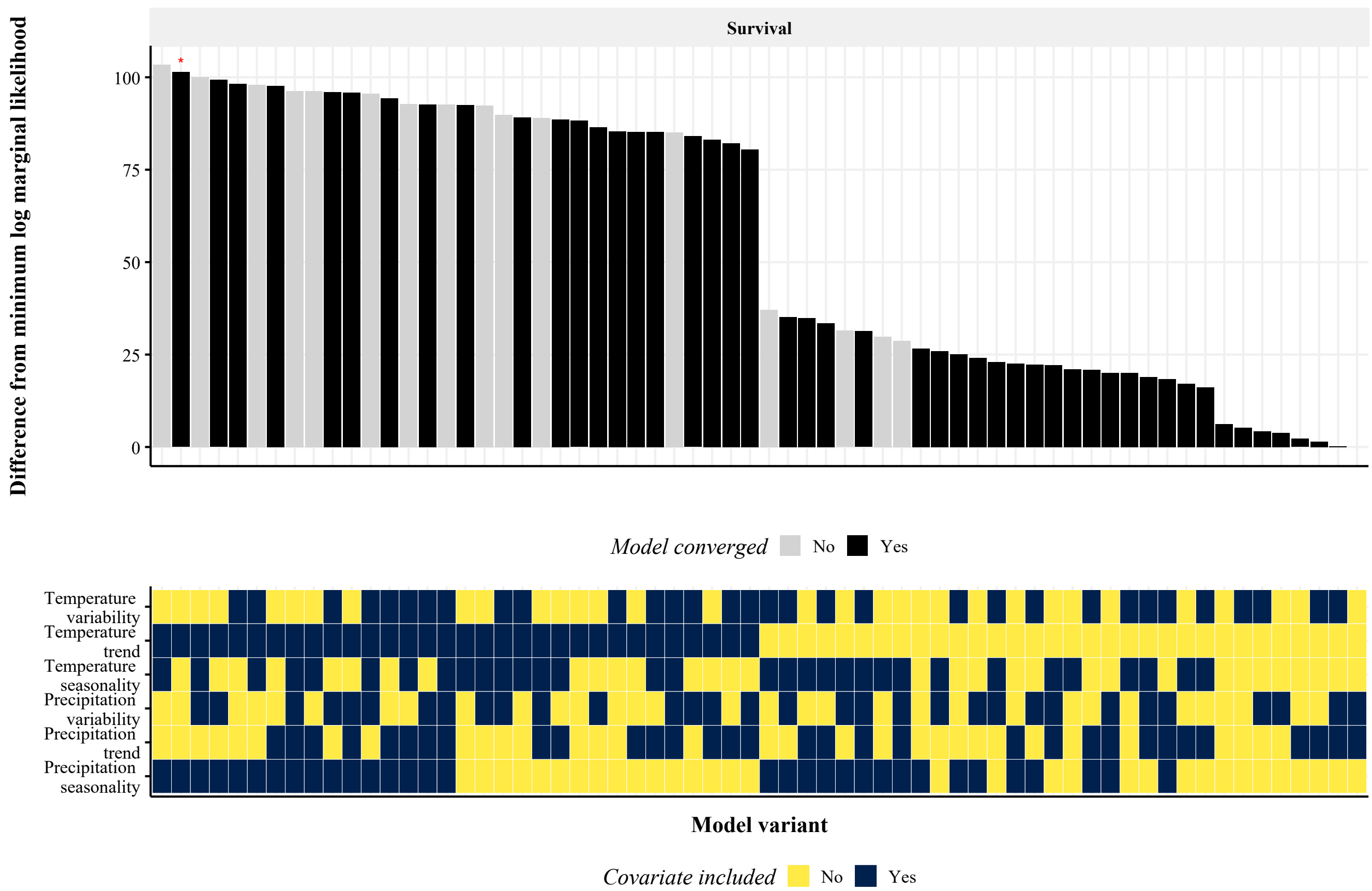

Fig. S3. Survival model performance for 64 model variants comprising all combinations of 6 climate predictors. The model estimates recapture at time $t + 1$ given demographic and climate covariates at time t. These estimates are subsequently adjusted to obtain probabilities of survival (Section F.4). Models were fit to three decades of rodent trapping data (3) and climate data (1, 2) using a No-U-Turn Sampler (8) Hamiltonian Monte Carlo algorithm in Stan (7), with prior distributions as specified in Table S1. Approximate marginal log-likelihoods were calculated via bridge sampling (10). The bottom panel shows the covariates included in each model, while the top panel shows the corresponding performance of that model relative to the worst-performing model. Model variants are ordered from best (left) to worst (right) performing. Grey bars indicate models that did not converge ($\hat{R} \geq 1.05$ for ≥ 1 parameter(s)). *Bayes factor ≥ 5 and < 10 compared to the best-performing model.

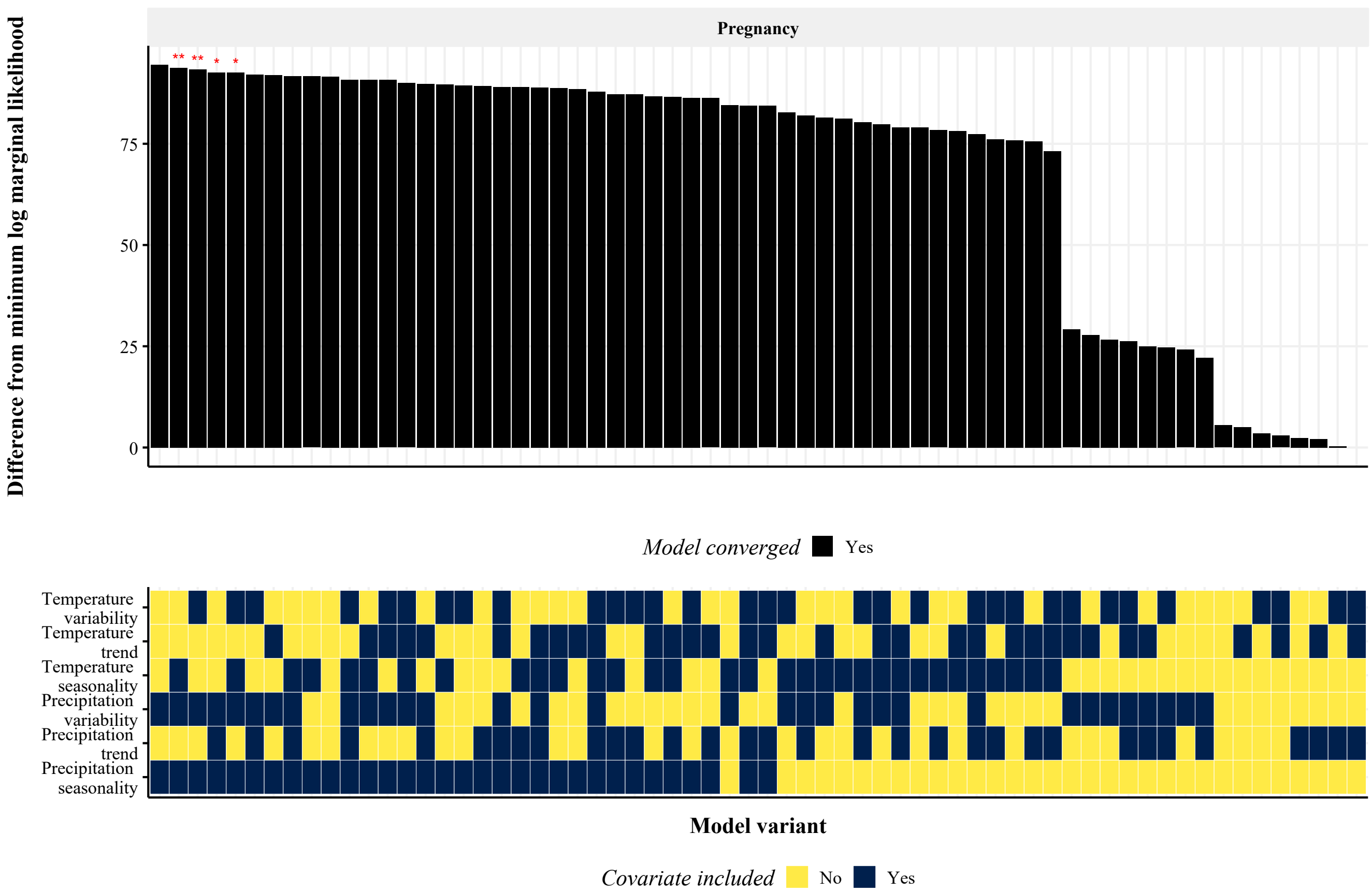

Fig. S4. Pregnancy model performance for 64 model variants comprising all combinations of 6 climate predictors. The model estimates pregnancy at time $t + 1$ given demographic and climate covariates at time t. Models were fit to three decades of rodent trapping data (3) and climate data (1, 2) using a No-U-Turn Sampler (8) Hamiltonian Monte Carlo algorithm in Stan (7), with prior distributions as specified in Table S1. Approximate marginal log-likelihoods were calculated via bridge sampling (10). The bottom panel shows the covariates included in each model, while the top panel shows the corresponding performance of that model relative to the worst-performing model. Model variants are ordered from best (left) to worst (right) performing. Grey bars indicate models that did not converge ($\hat{R} \geq 1.05$ for ≥ 1 parameter(s)). **Bayes factor < 5 compared to the best-performing model; *Bayes factor ≥ 5 and < 10 compared to the best-performing model.

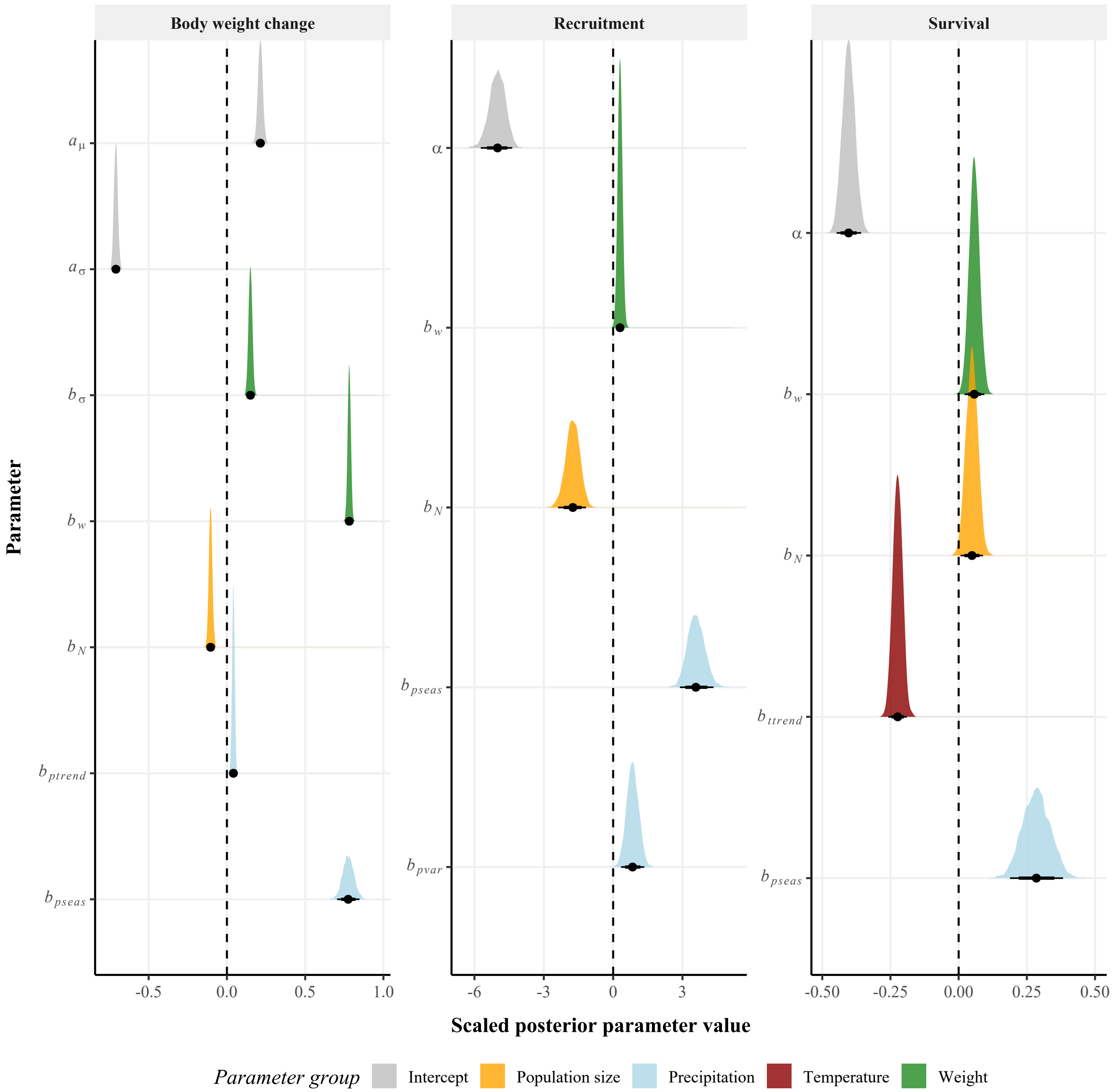

Fig. S5. Estimated posterior distributions of unit-scaled coefficients of demographic process models. The density of each posterior distribution is shown alongside a posterior median (point), 80% credible interval (thick horizontal line), and 95% credible interval (thin horizontal line). The vertical dashed line represents no effect. An approximate Bayesian approach was used to fit the model to rodent trapping data (3) and climate data (1, 2). The a parameters represent intercepts for: the Bernoulli processes of recruitment and survival (a), the mean of the body weight distribution (a_μ), and the standard deviation of the body weight distribution (a_σ). The b parameters represent coefficients for: the standard deviation of the body weight distribution (b_σ), body weight (b_w), population size (b_N), precipitation trend ($b_{p_{\text{trend}}}$), precipitation seasonality ($b_{p_{\text{seas}}}$), precipitation variability ($b_{p_{\text{var}}}$), and temperature trend ($b_{t_{\text{trend}}}$).

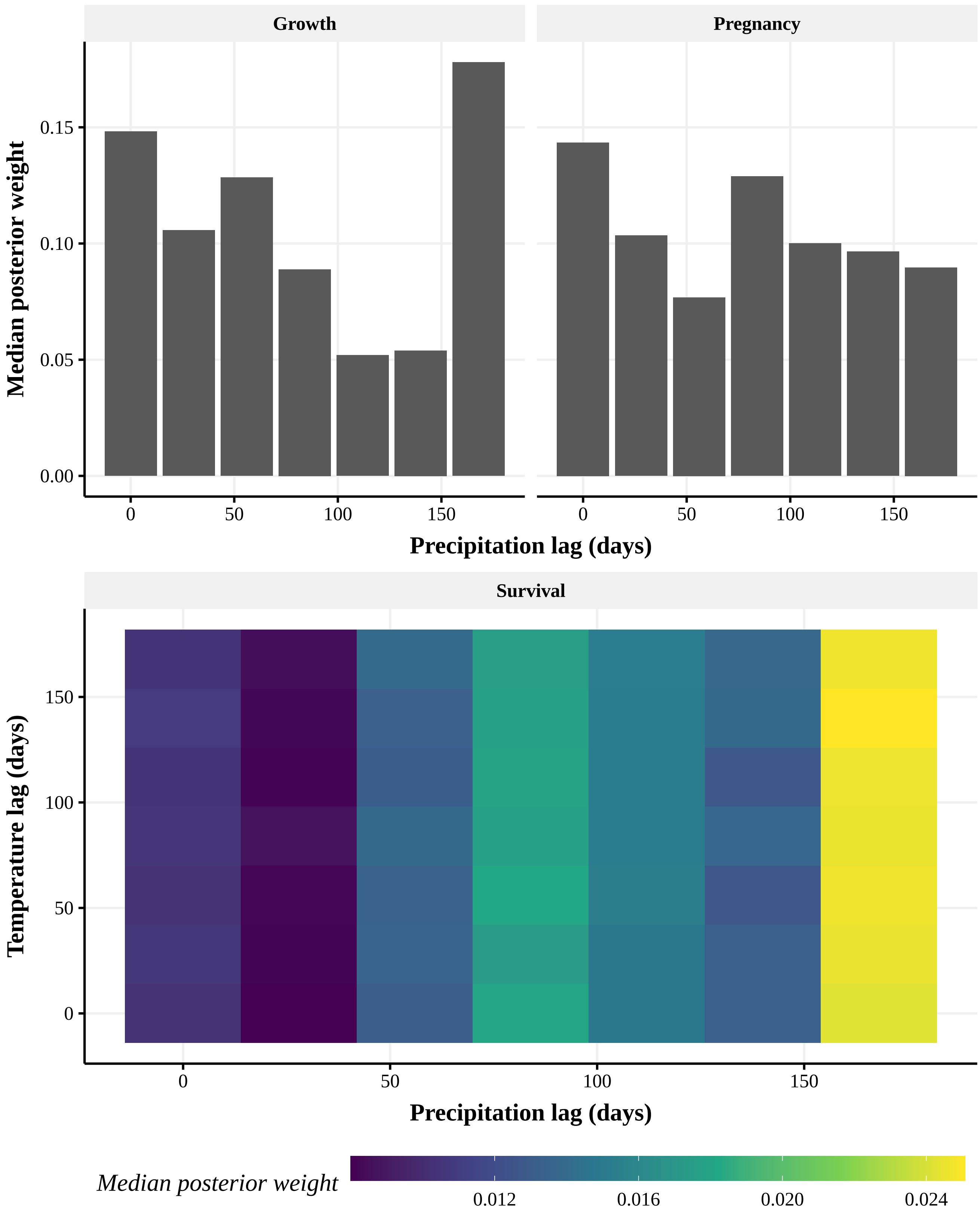

Fig. S6. Median posterior estimates of climate lag weights ($N = 4000$ posterior samples) for the best-performing models of growth, pregnancy, and survival. While growth and pregnancy models only included precipitation predictors, survival also included temperature predictors and hence for this model lag weight estimates are shown by precipitation and temperature lag. Models were fit to three decades of rodent trapping data (3) and climate data (1, 2) using a No-U-Turn Sampler (8) Hamiltonian Monte Carlo algorithm in Stan (7), with prior distributions as specified in Table S1.

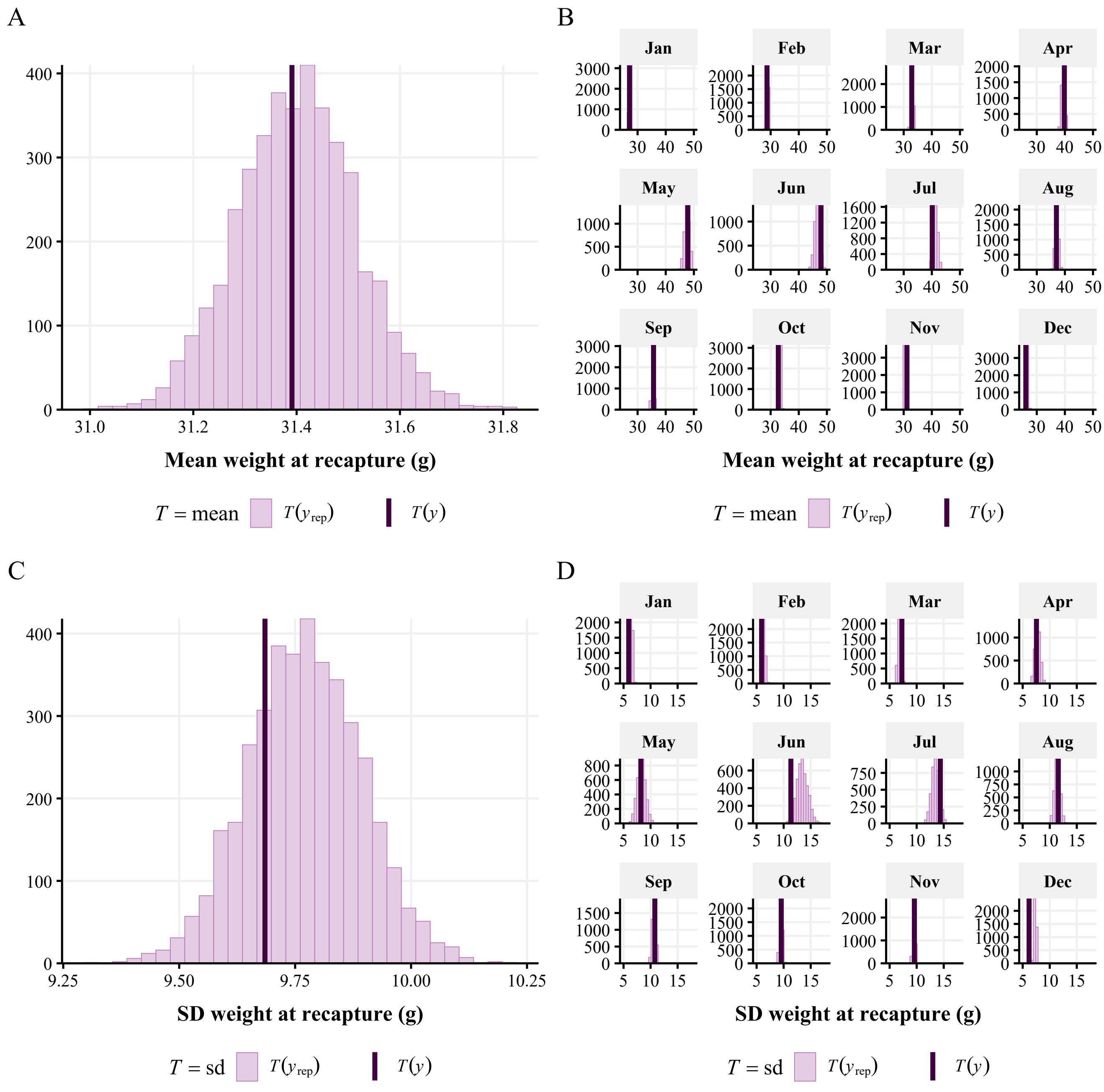

Fig. S7. Posterior predictive checks (PPCs) of the best-performing body weight change model. PPCs were performed by simulating 4000 posterior samples from the posterior predictive distribution of the fitted model to obtain predicted replications y_{rep} (pink histogram) of the observed data y (dark vertical line). Panels **A** and **B** show for the whole population and by month, respectively, the observed versus predicted values of the mean weight at time $t+1$. Panels **C** and **D** show for the whole population and by month, respectively, the observed versus predicted values of the standard deviation of weight at time $t+1$.

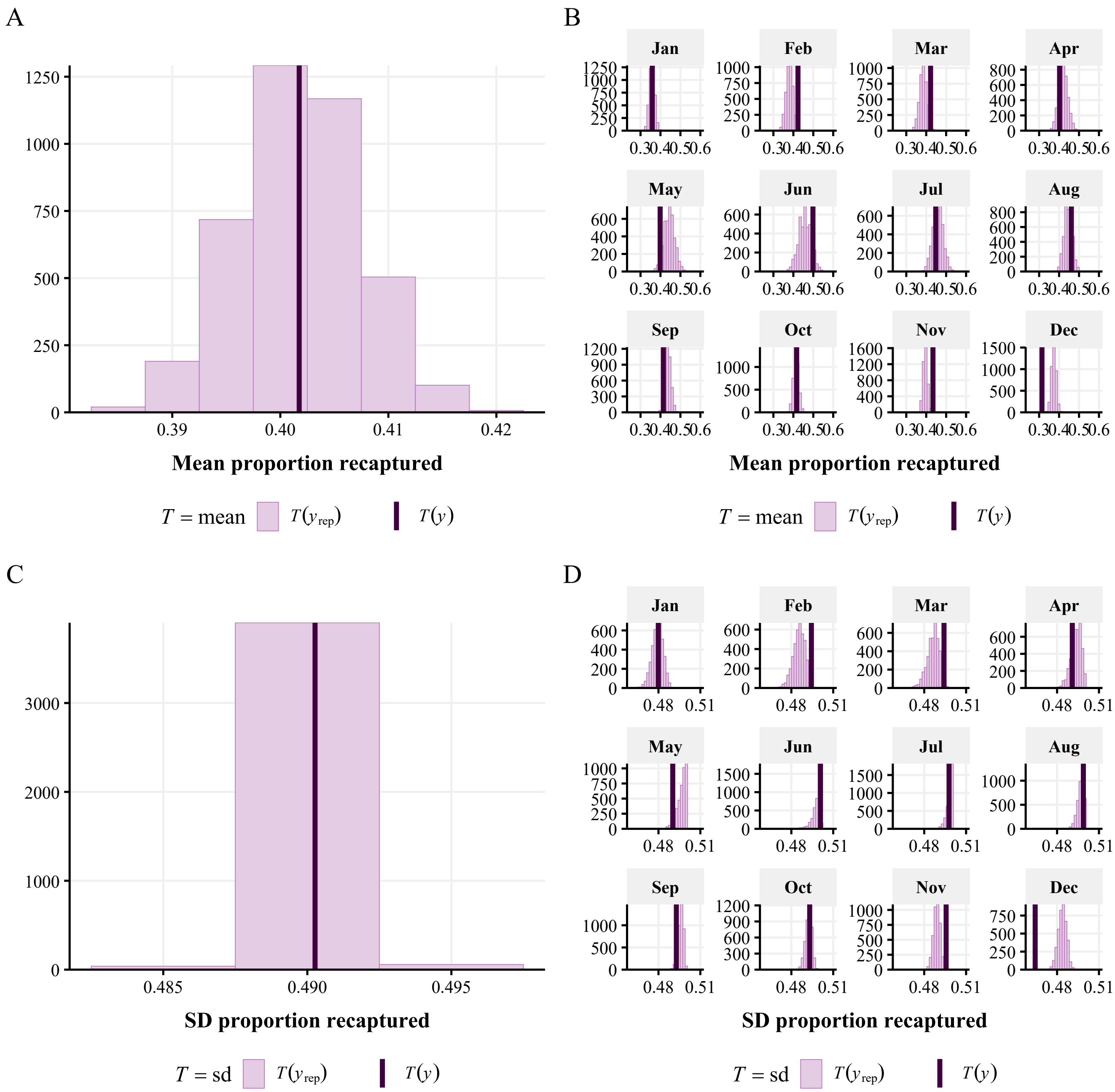

Fig. S8. Posterior predictive checks (PPCs) of the best-performing survival model. PPCs were performed by simulating 4000 posterior samples from the posterior predictive distribution of the fitted model to obtain predicted replications y_{rep} (pink histogram) of the observed data y (dark vertical line). Panels **A** and **B** show for the whole population and by month, respectively, the observed versus predicted values of the mean proportion of individuals recaptured at time $t+1$. Panels **C** and **D** show for the whole population and by month, respectively, the observed versus predicted values of the standard deviation of the proportion of individuals recaptured at time $t+1$.

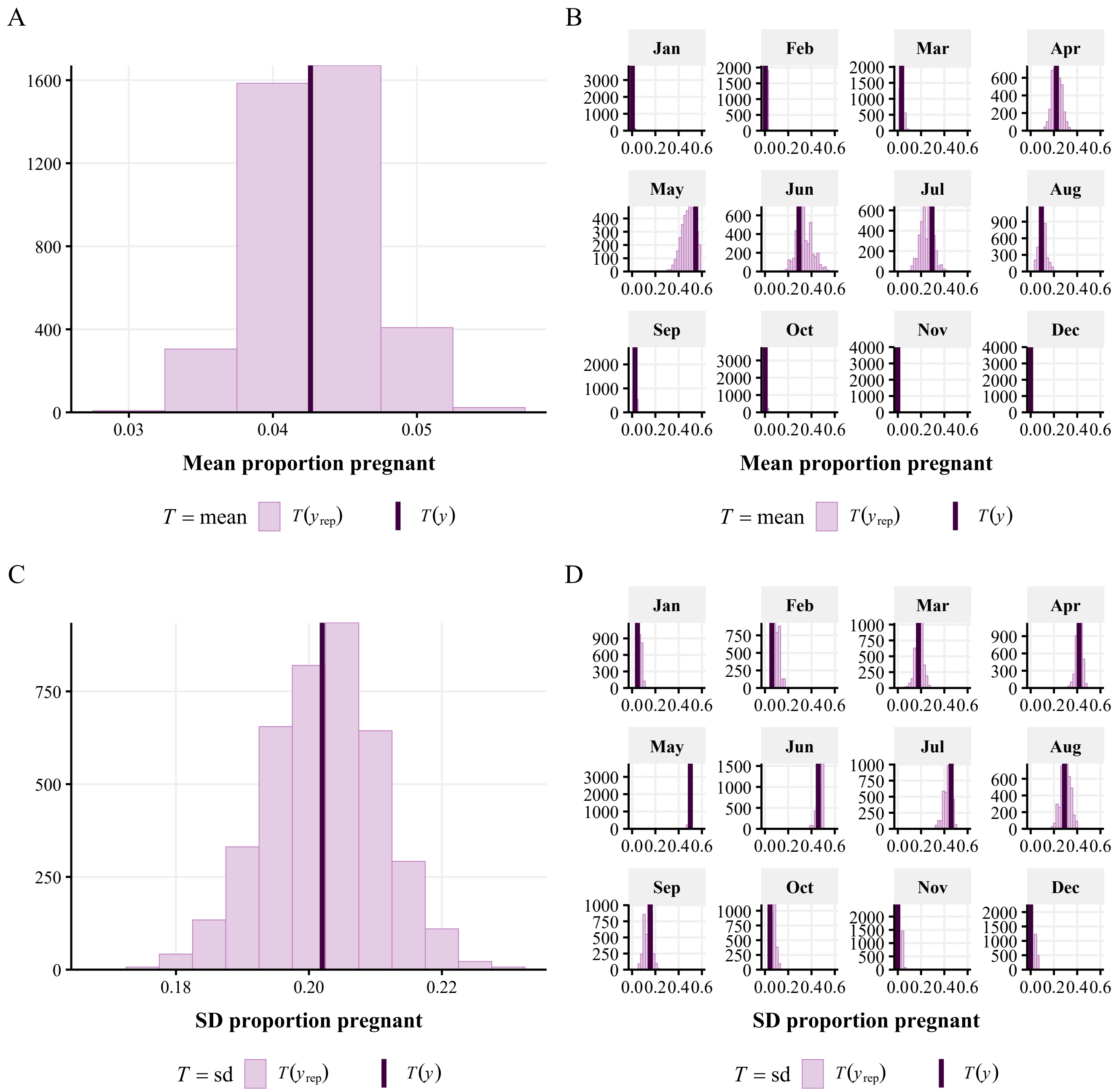

Fig. S9. Posterior predictive checks (PPCs) of the best-performing pregnancy model. PPCs were performed by simulating 4000 posterior samples from the posterior predictive distribution of the fitted model to obtain predicted replications y_{rep} (pink histogram) of the observed data y (dark vertical line). Panels **A** and **B** show for the whole population and by month, respectively, the observed versus predicted values of the mean proportion of individuals pregnant at time $t+1$. Panels **C** and **D** show for the whole population and by month, respectively, the observed versus predicted values of the standard deviation of the proportion of individuals pregnant at time $t+1$.

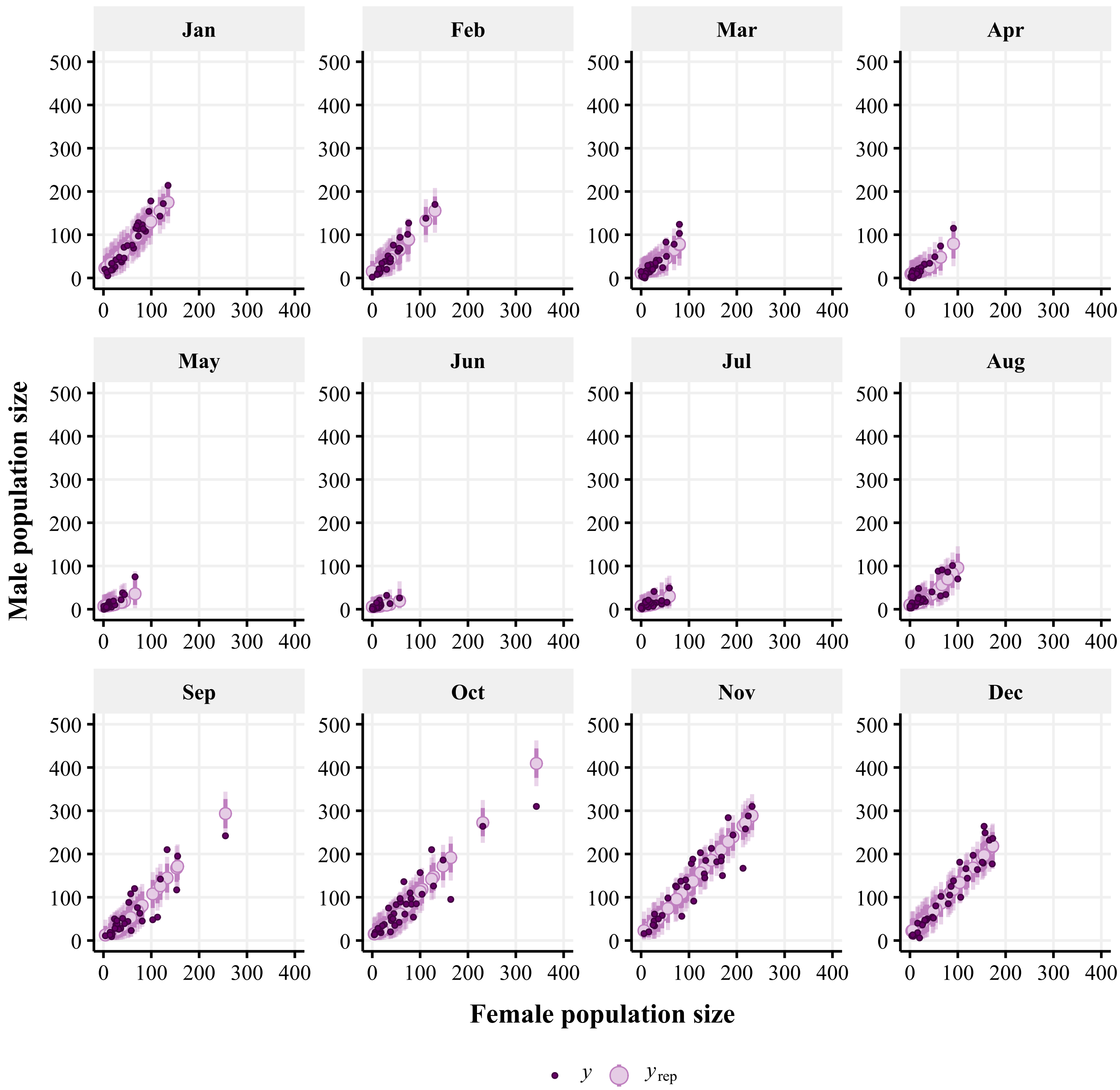

Fig. S10. Posterior predictive checks (PPCs) of the best-performing male population size model, which includes a coefficient for female population size and month as a random intercept. PPCs were performed by simulating 4000 posterior samples from the posterior predictive distribution of the fitted model to obtain predictions y_{rep} (pink points representing medians and inner and outer error bars 80% and 95% credible intervals, respectively) of the observed data y (dark points).

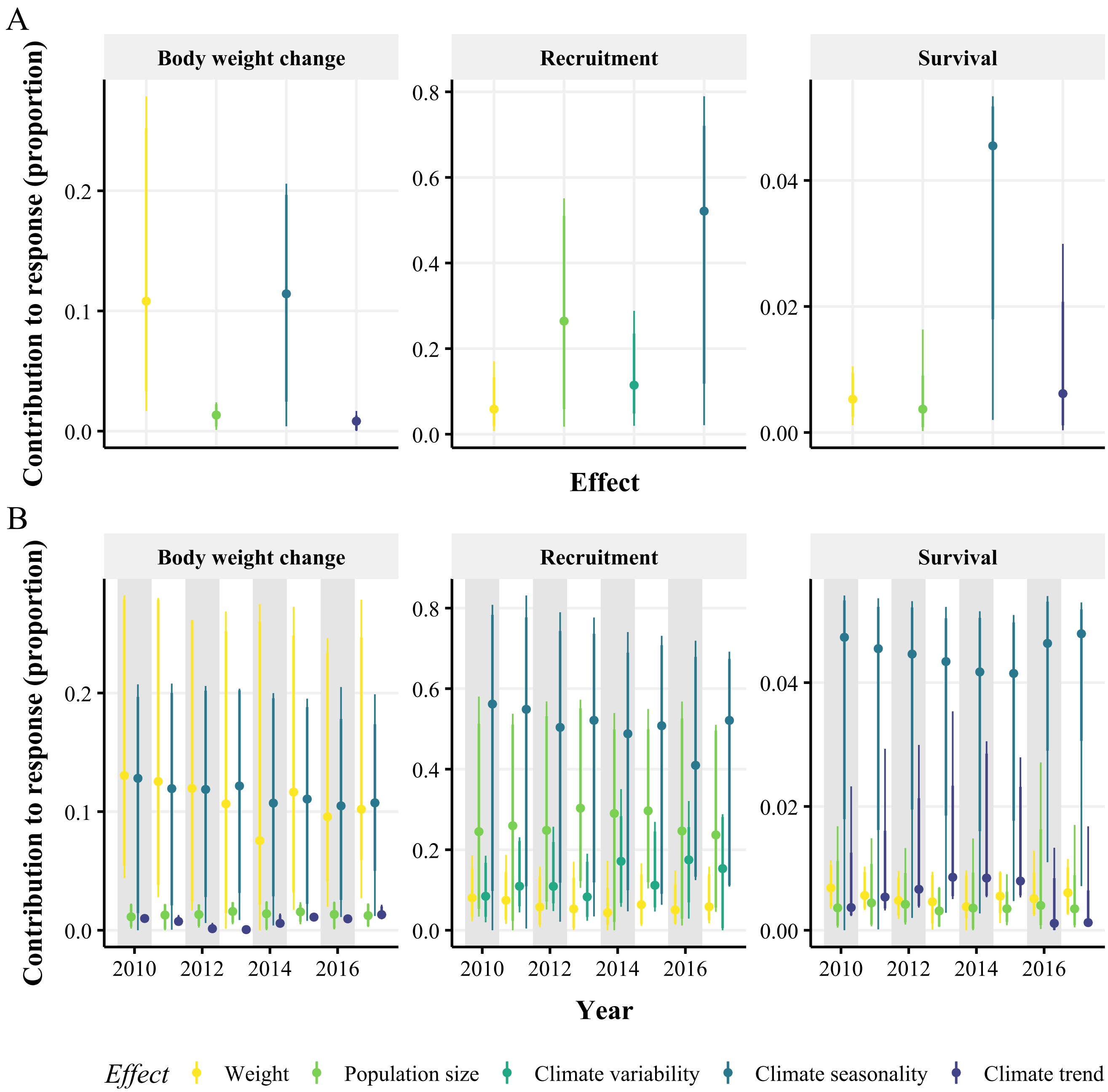

Fig. S11. Posterior predictions ($N = 1000$ simulations) of the contribution of each covariate to the modelled demographic process (shown as a proportion of the total response over time (**A**) and the year-specific proportion of the response (**B**)). The median prediction (point) is shown alongside the 80% credible interval (thick vertical line), and 95% credible interval (thin vertical line). An approximate Bayesian approach was used to fit the model to rodent trapping data (3), climate data (1, 2), and arenavirus serological data (14). Posterior predictive samples were obtained through Bayesian melding (Section H) (17, 18).

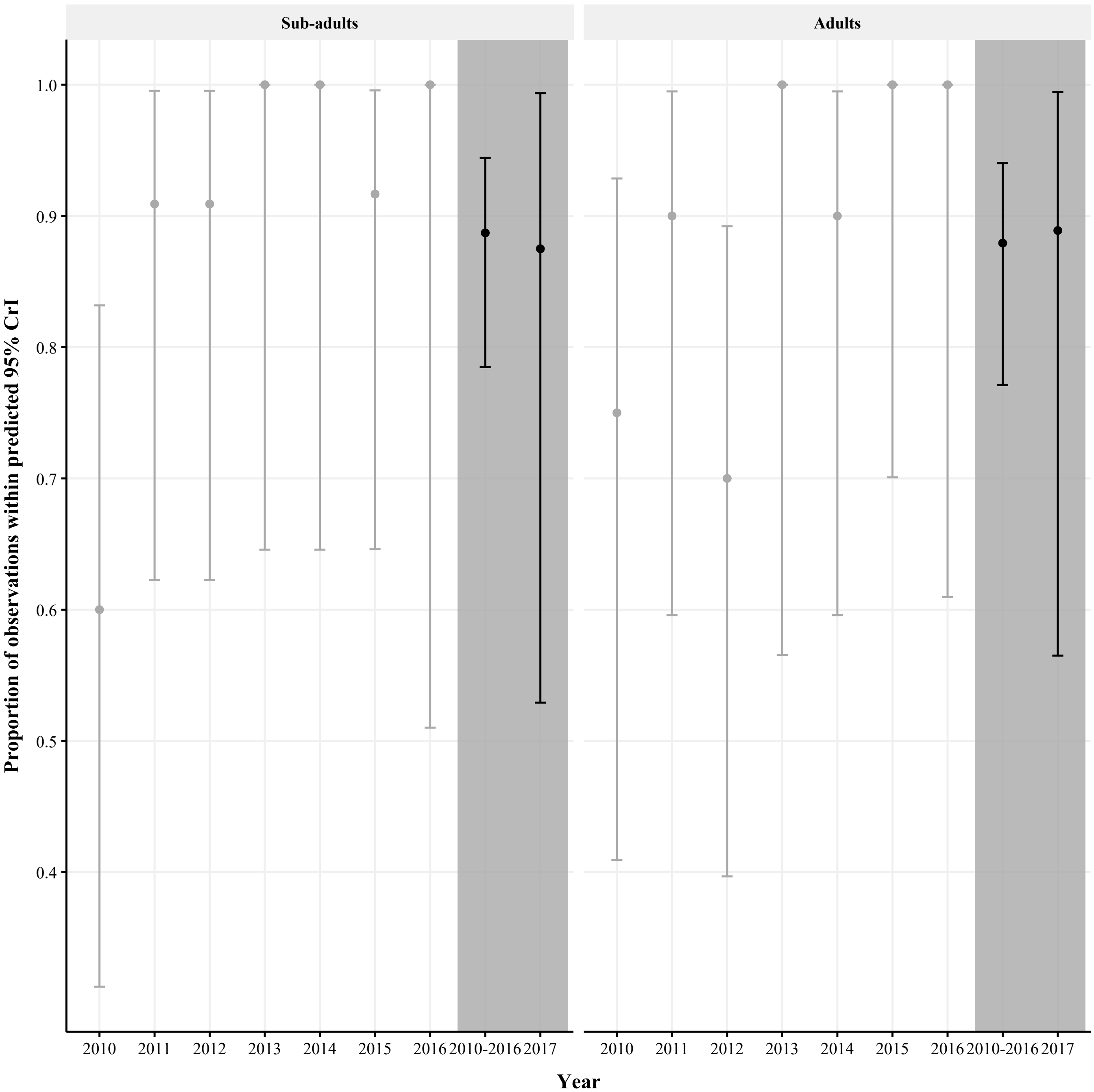

Fig. S12. Coverage analysis showing overlap between predicted seroprevalence 95% credible intervals (from $N = 1000$ posterior simulations) and observed seroprevalence 95% binomial confidence intervals (N=139 trapping sessions, 2010–2017 (14)). For each rodent age class (sub-adults and adults) and year of the training dataset (2010–2016) we calculated the proportion of trapping sessions for which observed seroprevalence 95% confidence intervals overlapped predicted seroprevalence 95% credible intervals (grey points), expressing uncertainty via Wilson binomial 95% confidence intervals (grey whiskers). The black points and whiskers in the grey box shows coverage estimates summarized across all training data years (2010–2016) versus estimates calculated for the test data year (2017). An approximate Bayesian approach was used to fit the model to rodent trapping data (3), climate data (1, 2), and arenavirus serological data (14). Posterior predictive samples were obtained through Bayesian melding (Section H) (17, 18).

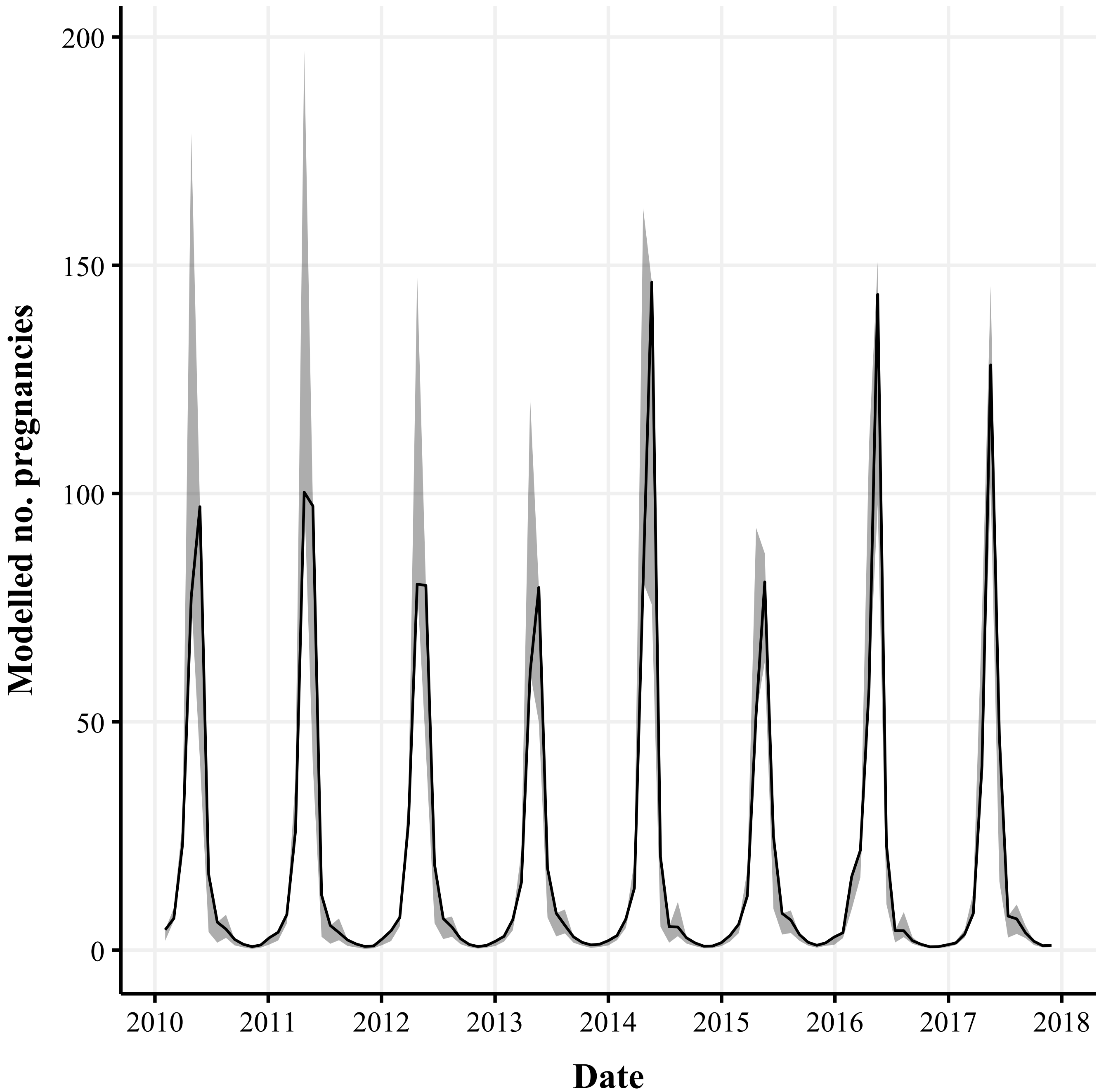

Fig. S13. Posterior predictions ($N = 1000$ simulations) of the predicted number of pregnant rodents. The line represents the median while shaded areas represent the 95% credible interval. An approximate Bayesian approach was used to fit the model to rodent trapping data (3), climate data (1, 2), and arenavirus serological data (14). Posterior predictive samples were obtained through Bayesian melding (Section H) (17, 18).

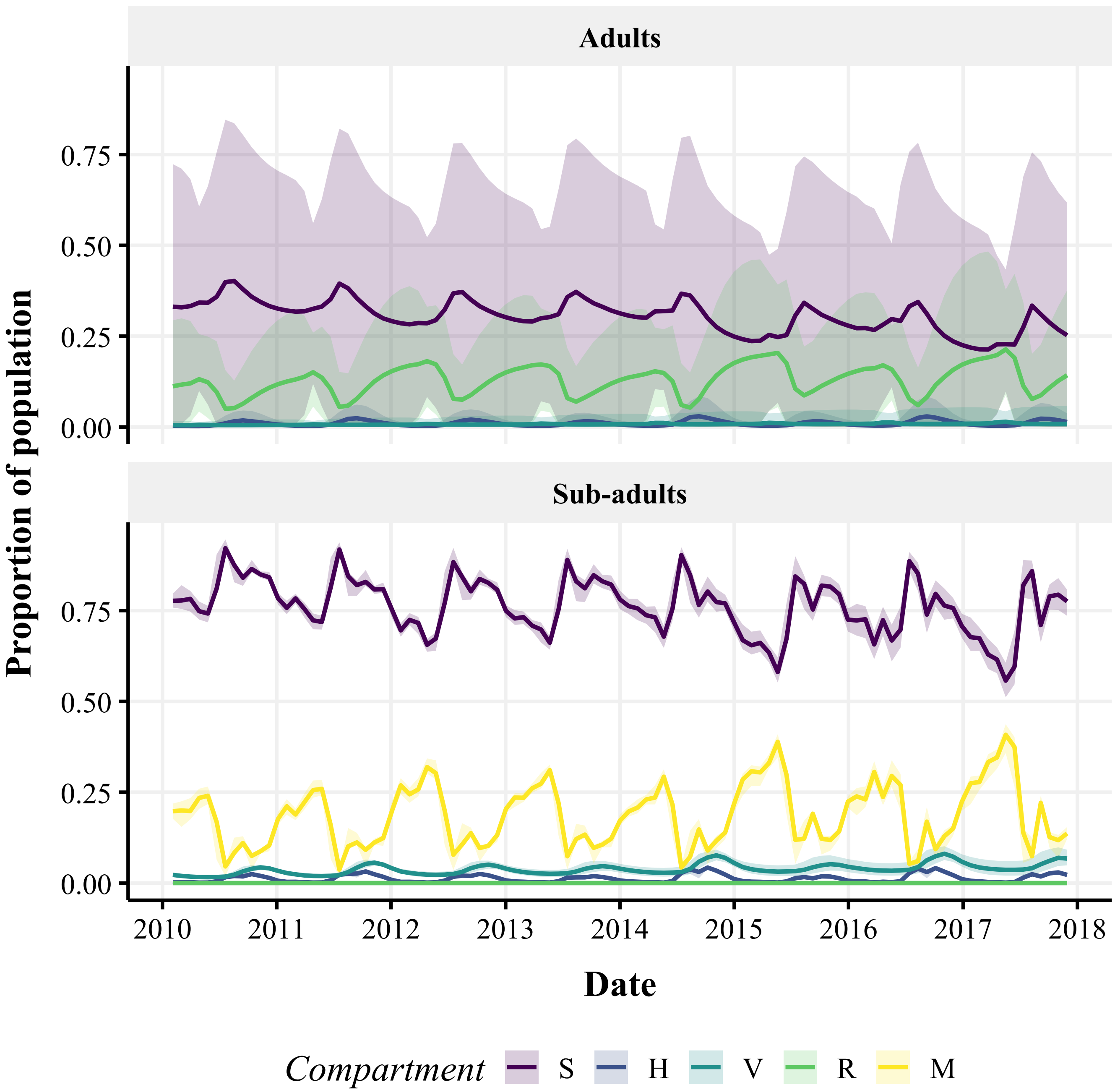

Fig. S14. Posterior predictions ($N = 1000$ simulations) of the proportion of the modelled population in each epidemiological compartment at a given time: susceptible (S), horizontally infected (H), vertically infected (V), recovered (R), and maternal antibody positive (M). The time-specific proportion of the population in each compartment was calculated for each model time-step by demographic group (sub-adult vs. adult) independently for each posterior simulation. Lines represent the medians while shaded areas represent 95% credible intervals. An approximate Bayesian approach was used to fit the model to rodent trapping data (3), climate data (1, 2), and arenavirus serological data (14). Posterior predictive samples were obtained through Bayesian melding (Section H) (17, 18).

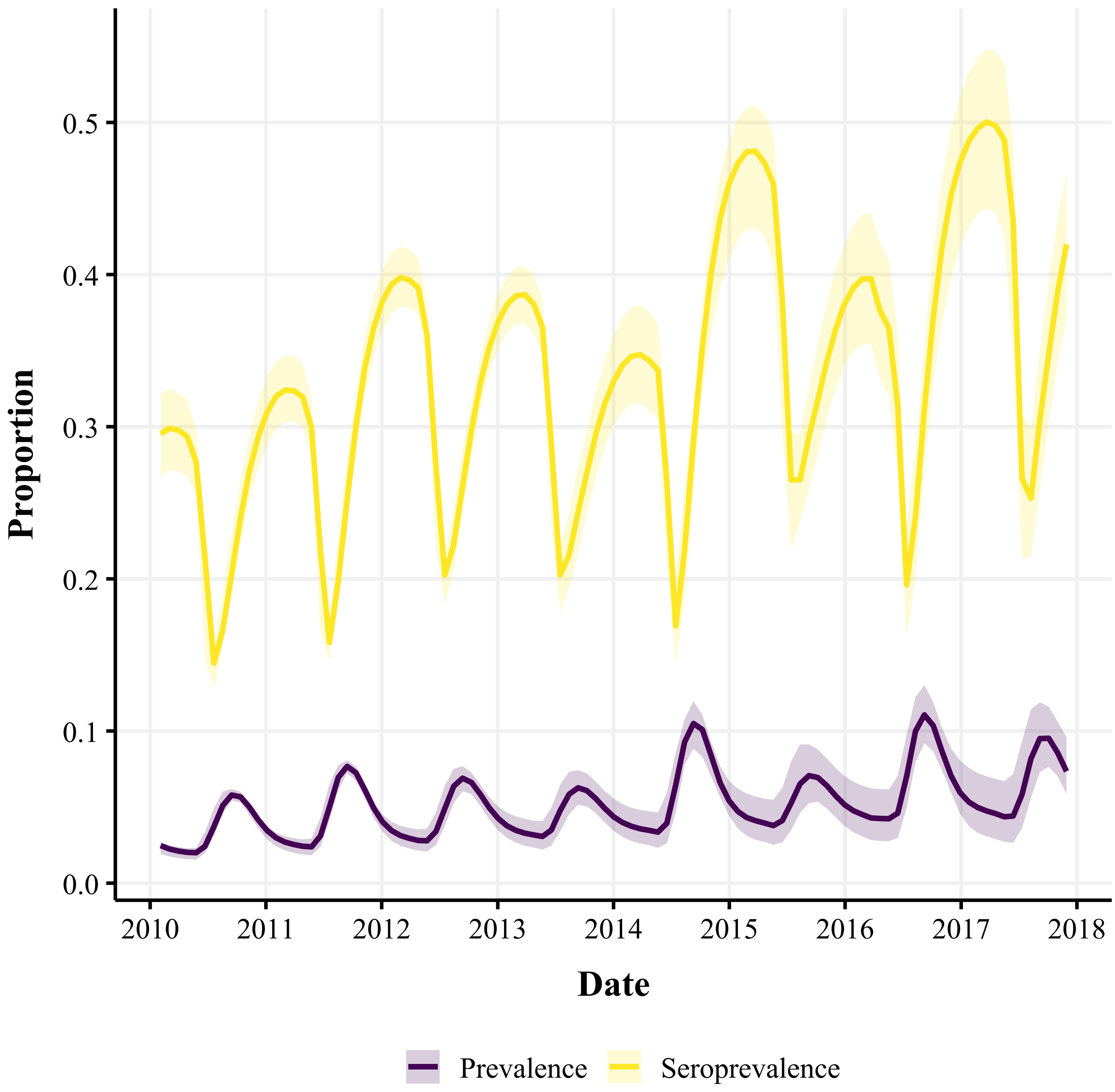

Fig. S15. Posterior predictions ($N = 1000$ simulations) of the relationship between prevalence and seroprevalence. Lines represent the medians while shaded areas represent 95% credible intervals. The time-specific prevalence was calculated as $[H(t) + V(t)]/N(t)$. The time-specific seroprevalence was calculated as $[N(t) - S(t)]/N(t)$ (Section G). An approximate Bayesian approach was used to fit the model to rodent trapping data (3), climate data (1, 2), and arenavirus serological data (14). Posterior predictive samples were obtained through Bayesian melding (Section H) (17, 18).

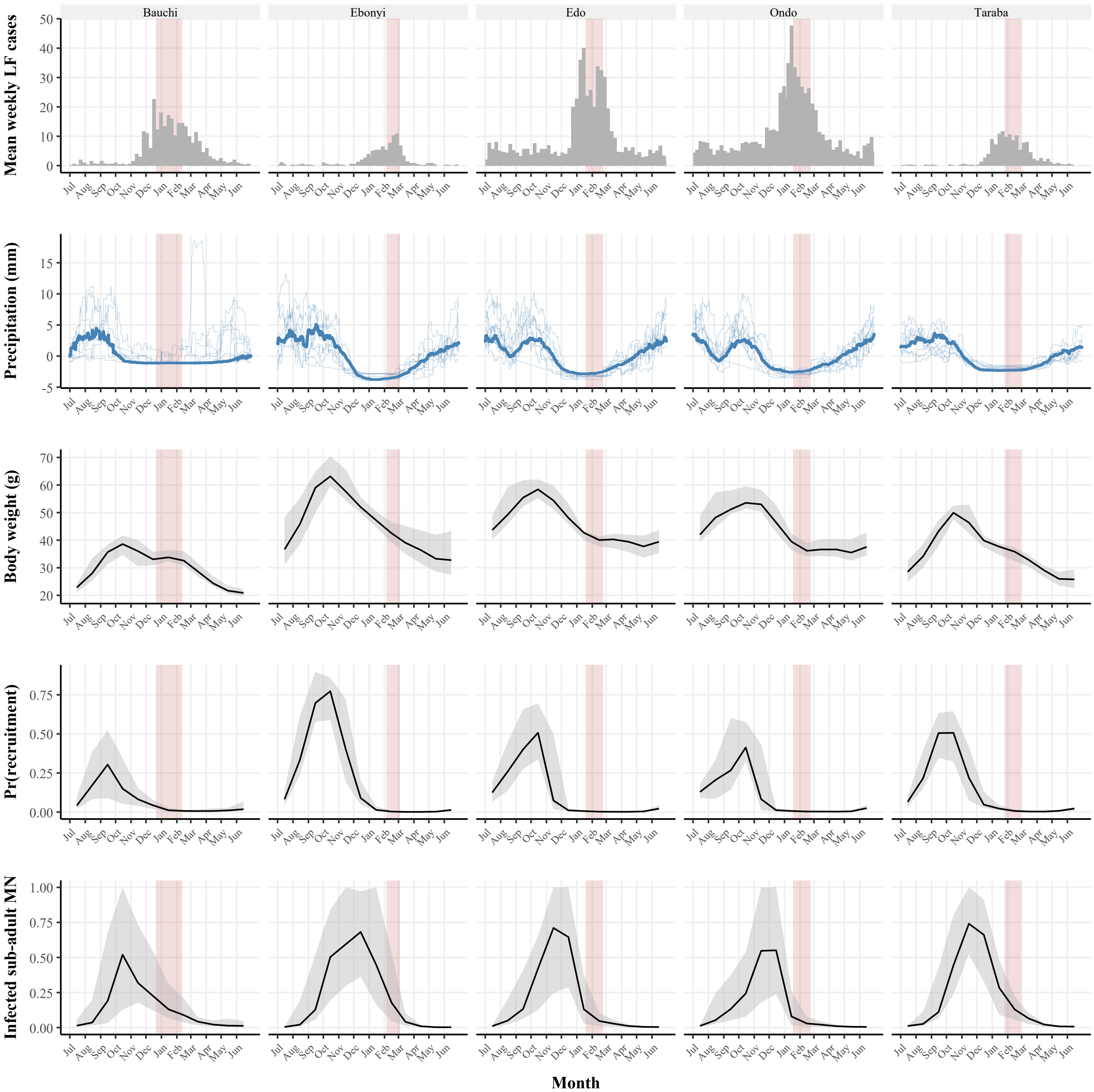

Fig. S16. Comparison for five Nigerian states of the seasonal pattern of Lassa fever (LF) outbreaks (top row) to those of the climatic and demographic drivers of *Mastomys natalensis* (MN) population dynamics (middle three rows), and of the model-predicted number of infected sub-adult MN (bottom row). The top row of panels shows weekly laboratory-confirmed LF cases averaged over 2018–2025 (19). In each panel, the red shaded area shows the interquartile range of peak LF outbreak timing for the given state. The second row of panels shows detrended precipitation data (1, 2) as the seasonal (dark blue line) and variable (light blue lines) components from a decomposed time series. The bottom three rows of panels (MN weight, probability of recruitment, and the relative number of infected sub-adult MN) show the median (black line) and 95% credible interval (grey shaded area) from $N = 1000$ posterior predictive simulations over 2018–2025. For each simulation, weight and P(recruitment) were calculated as time-specific means across the population. The number of infected sub-adult MN was calculated as $H(t) + V(t)$ and made relative to the maximum value in each simulation before calculating summarized values across all simulations. An approximate Bayesian approach was used to fit the model to rodent trapping data (3), climate data (1, 2), and arenavirus serological data (14). Posterior predictive samples were obtained through Bayesian melding (Section H) (17, 18).

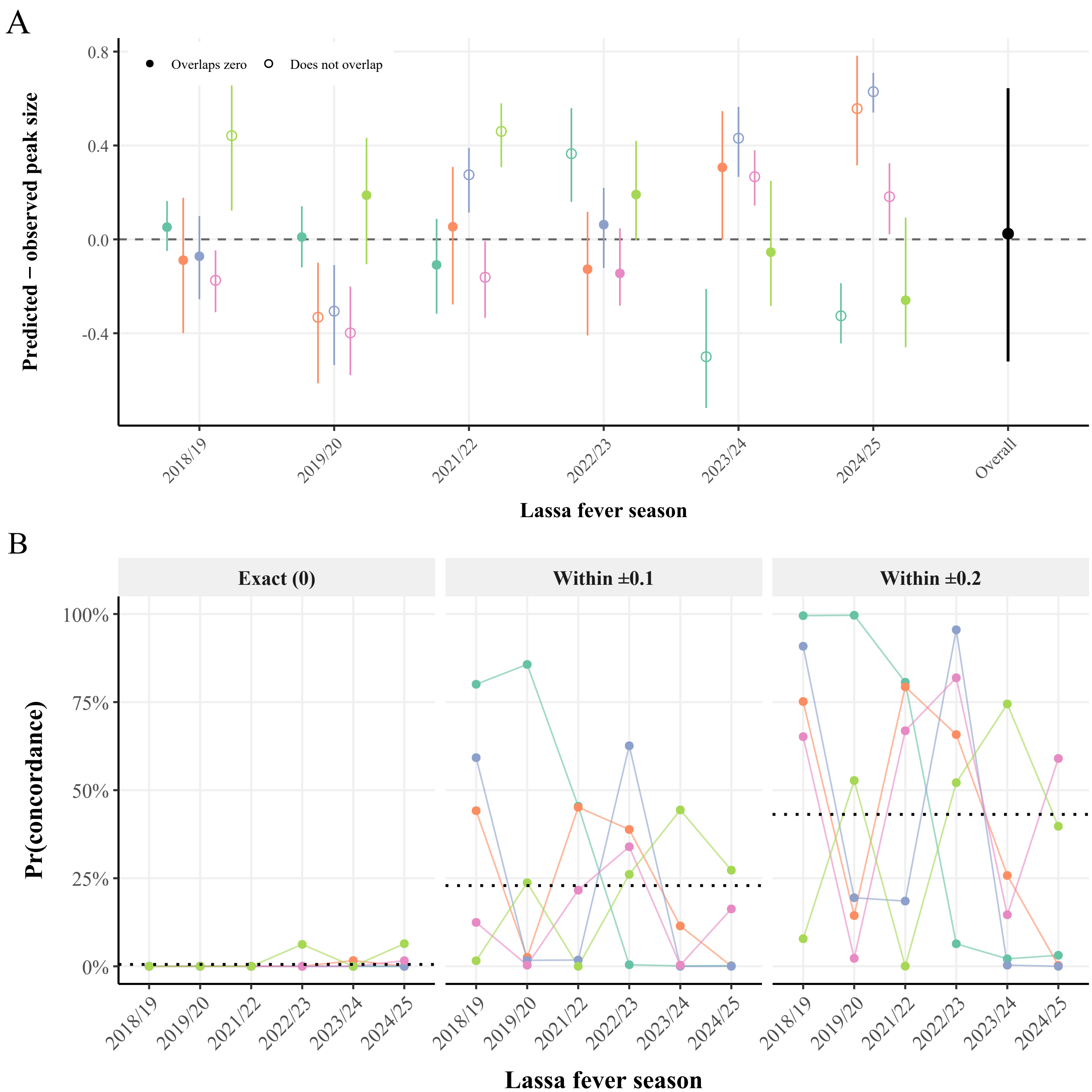

Fig. S17. (***A***) Differences in peak size (relative units) between the predicted number of infected sub-adult rodents and observed LF outbreaks (19) by state and LF season, with the overall estimate (black) pooled across all states and seasons. Points represent medians and error bars 95% credible intervals. The dashed horizontal lines indicate perfect agreement. Peak size comparisons used N=10,000 bootstrap samples drawn independently from model and observation posterior distributions. Uncertainty in reported LF cases was represented by N=1,000 Poisson-distributed realizations of weekly case counts; estimated infection dates were obtained by convolving each realization with a LF incubation period distribution (sampling N=1,000 random parameter sets from the incubation period posterior; see Section I.2 and Table S3). (***B***) Probability of concordance between predicted and observed peak sizes across three agreement thresholds — exact agreement, within ±0.1 relative units, and within ±0.2 relative units — by state and season. Dotted horizontal lines show the overall probability of concordance at each threshold, pooled across all states and seasons.

References

1. National Oceanic and Atmospheric Administration, Climate Prediction Centre global daily precipitation (2025).
2. National Oceanic and Atmospheric Administration, Climate Prediction Centre global daily temperature (2025).
3. H Leirs, et al., Twenty-nine years of continuous monthly capture-mark-recapture data of multimammate mice (Mastomys natalensis) in Morogoro, Tanzania. *Sci. Data* **10**, 1–8 (2023).
4. National Oceanic and Atmospheric Administration, Climate Prediction Centre global temperature long-term mean 1991-2020 (2025).
5. RB Cleveland, WS Cleveland, JE McRae, I Terpenning, STL: A seasonal-trend decomposition procedure based on LOESS. *J. Off. Stat.* **6**, 3–73 (1990).
6. J Meester, Early post-natal development of multi-mammate mice Rattus (Mastomys) natalensis (A. Smith). *Annals Transvaal Mus.* **24** (1960).
7. B Carpenter, et al., Stan: A Probabilistic Programming Language. *J. Stat. Softw.* **76**, 1–32 (2017).
8. MD Hoffman, A Gelman, The No-U-Turn sampler: adaptively setting path lengths in Hamiltonian Monte Carlo. *J. Mach. Learn. Res.* **15**, 1593–1623 (2014).
9. PC Bürkner, brms: An R Package for Bayesian Multilevel Models Using Stan. *J. Stat. Softw.* **80**, 1–28 (2017).
10. QF Gronau, H Singmann, EJ Wagenmakers, bridgesampling: An R Package for Estimating Normalizing Constants. *J. Stat. Softw.* **92**, 1–29 (2020).
11. TP Jackson, RJV Aarde, Sex- and Species-Specific Growth Patterns in Cryptic African Rodents, Mastomys natalensis and M. coucha. *J. Mammal.* **84**, 851–860 (2003).
12. HL Johnston, WD Oliff, The oestrous cycle of female Rattus (Mastomys) natalensis (Smith) as observed in the laboratory. *Proc. Zool. Soc.* **124**, 605–613 (1954).
13. B Borremans, et al., Shedding dynamics of Morogoro virus, an African arenavirus closely related to Lassa virus, in its natural reservoir host Mastomys natalensis. *Sci. Reports* **5**, 1–8 (2015).
14. J Mariën, et al., Density dependence and persistence of Morogoro arenavirus transmission in a fluctuating population of its reservoir host. *J. Animal Ecol.* **89**, 506–518 (2020).
15. E Fichet-Calvet, E Lecompte, L Koivogui, S Daffis, JT Meulen, Reproductive Characteristics of Mastomys natalensis and Lassa Virus Prevalence in Guinea, West Africa. *Vector-Borne Zoonotic Dis.* **8**, 41–48 (2008).
16. RS John, HO Fatoyinbo, DTS Hayman, Modelling Lassa virus dynamics in West African Mastomys natalensis and the impact of human activities. *J. Royal Soc. Interface* **21** (2024).
17. D Poole, AE Raftery, Inference for Deterministic Simulation Models: The Bayesian Melding Approach. *J. Am. Stat. Assoc.* **95**, 1244–1255 (2000).
18. L Alkema, AE Raftery, SJ Clark, Probabilistic projections of HIV prevalence using Bayesian melding. *Annals Appl. Stat.* **1**, 229–248 (2007).
19. Nigeria Centre for Disease Control and Prevention, An update of Lassa fever outbreak in Nigeria (2025).
20. EA Ilori, et al., Increase in Lassa Fever Cases in Nigeria, January-March 2018. *Emerg. Infect. Dis.* **25**, 1026 (2019).
21. JD Frame, JM Baldwin, DJ Gocke, JM Troup, Lassa fever, a new virus disease of man from West Africa. I. Clinical description and pathological findings. *The Am. journal tropical medicine hygiene* **19**, 670–676 (1970).
22. PE Mertens, R Patton, JJ Baum, TP Monath, Clinical presentation of Lassa fever cases during the hospital epidemic at Zorzor, Liberia, March-April, 1972. *Am. J. Trop. Medicine Hyg.* **22** (1973).
23. SP Fisher-Hoch, et al., Review of cases of nosocomial Lassa fever in Nigeria: The high price of poor medical practice. *BMJ* **311**, 857 (1995).
24. NA Ajayi, et al., Containing a Lassa fever epidemic in a resource-limited setting: Outbreak description and lessons learned from Abakaliki, Nigeria (January-March 2012). *Int. J. Infect. Dis.* **17**, e1011–e1016 (2013).
25. A Yadouleton, et al., Lassa fever in Benin: description of the 2014 and 2016 epidemics and genetic characterization of a new Lassa virus. *Emerg. Microbes Infect.* **9**, 1761–1770 (2020).
26. CC Dan-Nwafor, et al., A cluster of nosocomial Lassa fever cases in a tertiary health facility in Nigeria: Description and lessons learned, 2018. *Int. J. Infect. Dis.* **83**, 88–94 (2019).
27. ECDC, Rapid risk assessment: Cases of Lassa fever in the Netherlands ex Sierra Leone (2019).
